# BCL-XL drives fibrotic and leukemic progression in myeloproliferative neoplasms

**DOI:** 10.3389/fimmu.2026.1818806

**Published:** 2026-06-02

**Authors:** Chunyan Wu, Yiting Wang, Quanchao Zhang, Chan Li, Yuanzhong Chen, Yong Wu

**Affiliations:** 1 Department of Hematology, Fujian Medical University Union Hospital, Fujian Medical University, Fuzhou, Fujian, China; 2 Fujian Institute of Hematology, Fujian Provincial Key Laboratory on Hematology, Fujian Medical University Union Hospital, Fuzhou, Fujian, China; 3 Department of Hematology, Heping Hospital Affiliated to Changzhi Medical College, Changzhi Medical College, Changzhi, Shanxi, China

**Keywords:** apoptosis resistance, Bcl-XL, fibrosis, mesenchymal stem cells, myeloproliferative neoplasms

## Abstract

**Background:**

Myeloproliferative neoplasms (MPNs) are frequently accompanied by bone marrow fibrosis and leukemic transformation, yet the cellular and molecular mechanisms that sustain apoptosis resistance and fibrotic progression remain unclear.

**Methods:**

Bone marrow mesenchymal stromal cells (BM-MSCs) from patients with polycythemia vera (PV), essential thrombocythemia (ET), and primary myelofibrosis (PMF) were analyzed for fibrotic phenotype, apoptosis signaling, and pathway activation using cell counting kit-8, immunofluorescence, flow cytometry, western blotting, hydroxyproline and transmission electron microscopy. The cytotoxic and antifibrotic effects of the BCL-XL inhibitor ABT-263 (navitoclax), alone or combined with the JAK2 inhibitor ruxolitinib, were evaluated in stromal and hematopoietic contexts.

**Results:**

BCL-XL was markedly upregulated in JAK2-driven disease and predominated over other BCL-2 family members in both malignant hematopoietic cells and fibrotic stromal compartments. MSCs derived from MPN patients exhibited a myofibroblast-like phenotype characterized by increased α-smooth muscle actin (α-SMA) and fibronectin (FN) expression. Pharmacologic inhibition of BCL-XL with ABT-263 selectively induced mitochondrial apoptosis in PMF-derived MSCs and attenuated their profibrotic features. Mechanistically, transforming growth factor β (TGF-β) activated both SMAD3 and STAT3 signaling in MSCs, indicating cooperative engagement of TGF-β/SMAD3 and JAK2/STAT3 pathways in stromal fibrotic activation. Combined inhibition of BCL-XL and JAK2 produced synergistic antifibrotic and pro-apoptotic effects in MSCs, post-MPN acute myeloid leukemia (AML) cell lines, and patient-derived cells resistant to ruxolitinib.

**Conclusion:**

Collectively, these findings identified BCL-XL as a key mediator of MPN-associated fibrosis and therapeutic resistance, and confirmed dual targeting of BCL-XL and JAK2 as a rational strategy for advanced MPN.

## Introduction

Myeloproliferative neoplasms (MPNs) are chronic clonal disorders of hematopoietic stem cells driven primarily by mutations of JAK2, CALR or MPL, all of which converge on constitutive activation of the JAK–STAT signaling cascade (1, 2). Philadelphia chromosome–negative MPNs include polycythemia vera (PV), essential thrombocythemia (ET), and primary myelofibrosis (PMF). Clinically, patients present with splenomegaly, constitutional symptoms, and thromboembolic complications, and with disease progression, may evolve to advanced myelofibrosis (MF) or secondary acute myeloid leukemia (sAML), aggressive disease stages characterized by poor clinical prognosis and limited therapeutic modalities (3–5). The JAK1/2 inhibitor ruxolitinib remains the cornerstone of therapy, providing symptomatic and survival benefit (6). However, it cannot eradicate the malignant clone or reverse bone marrow fibrosis, and responses in accelerated or blast-phase MPN are transient (7). These limitations highlight the urgent need to identify complementary molecular targets capable of modifying disease biology beyond JAK inhibition.

Dysregulation of apoptosis constitutes a critical feature of MPN pathogenesis (1, 8). Members of the BCL-2 family regulate mitochondrial integrity via a dynamic balance of pro-apoptotic and anti-apoptotic proteins (9). Among these, BCL-XL plays a pivotal role in megakaryocyte maturation and platelet survival (10). Driver mutations of MPN result in constitutive activation of the cytokine receptor (11) and the JAK/STAT (12), MAPK (13), and PI3K/AKT (14) pathways. Aberrant activation of STAT3/5, a major effector of mutant JAK2 signaling, transcriptionally induces BCL-XL, conferring broad apoptotic resistance (15, 16). Previous studies have demonstrated that BCL-XL expression is markedly elevated in peripheral blood leukocytes of MF patients, correlating with disease severity and therapeutic resistance (8, 17–19); however, its expression pattern and biological function within the bone marrow microenvironment remain largely undefined. Recent work has further shown that BCL-XL expression increases along the clinical continuum from essential thrombocythemia to myelofibrosis, independently of JAK2 mutation status, and that pharmacologic inhibition of BCL-XL synergizes with ruxolitinib to trigger apoptosis in JAK2-mutant cells (8). These findings underscore BCL-XL as a lineage-restricted survival determinant within the JAK2/STAT5 axis and suggest that its blockade may augment current JAK-targeted therapies.

Beyond the malignant clone, MPN progression is characterized by progressive bone marrow fibrosis, largely driven by abnormal megakaryocyte–stromal interactions and macrophage-mediated inflammatory signaling, as well as profibrotic cytokines including transforming growth factor β (TGF-β) (20). Mesenchymal stromal cells (MSCs) are pivotal mediators of fibrotic remodeling via the deposition of extracellular matrix proteins, which include as fibronectin (FN) and α-smooth muscle actin (α-SMA) (21). Nonetheless, the molecular processes that underpin MSC activation and fibrosis are not fully elucidated. Recent research suggests that anti-apoptotic molecules, such as BCL-XL, may safeguard myofibroblastic cells against apoptosis, thus perpetuating fibrosis (22). Investigating the noncanonical role of BCL-XL in the stromal compartment may reveal new understanding of the interaction between clonal and microenvironmental elements of MPN.

Translational initiatives aimed at targeting BCL-XL have progressed to clinical assessment. The BH3 mimetic navitoclax (ABT-263), which suppresses BCL-2 and BCL-XL, has shown promising efficacy when combined with ruxolitinib in myelofibrosis patients, resulting in reductions in spleen enlargement, symptom burden, and histologic regression of marrow fibrosis (7). Nevertheless, on-target thrombocytopenia remains a major challenge due to the physiological role of BCL-XL in platelets (23). Next-generation medicines, including specific BCL-XL degraders (e.g., DT2216), are now being developed to mitigate this toxicity while maintaining anticancer efficacy, underscoring the increasing translational momentum surrounding this target (24).

This study investigated the biological and therapeutic significance of BCL-XL in the malignant cells and stromal components of myeloproliferative neoplasms (MPN). By integrating analyses from patient samples, MPN/AML cell lines, and publicly available transcriptomic datasets, we elucidated the expression patterns and functional implications of BCL-XL inhibition. Our findings demonstrated that BCL-XL was significantly upregulated in MPN, and targeted inhibition of BCL-XL markedly enhanced apoptosis in JAK2-mutant tumor cells and bone marrow stromal cells, thereby suppressing the progression of leukemia and fibrosis. These results highlighted the dual role of BCL-XL in promoting leukemia cell survival and sustaining stromal fibrosis, providing a molecular and translational rationale for a therapeutic strategy that simultaneously targets both BCL-XL and JAK2 in MPN.

## Materials and methods

### Cell lines and cell culture

The authenticity of all cell lines was confirmed by short tandem repeat (STR) analysis, and all cultures tested negative for mycoplasma contamination before experimental use. Human leukemia cell lines, including MV4-11, Kasumi, MOLM-13, MEG-01, HL60, and HEL, were maintained in RPMI-1640 medium supplemented with 10% fetal bovine serum (FBS). SET-2 cells were maintained in RPMI-1640 medium supplemented with 20% FBS. UKE-1 cells were grown in IMDM supplemented with 10% FBS, 1% Glutamax, and 10% horse serum. The UKE-1 and SET-2 cell lines were kindly provided by Professor Zhijian Xiao and Professor Bing Li from the Institute of Hematology, Chinese Academy of Medical Sciences. Cells were cultured at 37 °C in a humidified atmosphere containing 5% CO_2_. Cells in the logarithmic growth phase were used for subsequent experiments.

### Patients and healthy donors sample isolation

The research involving human subjects was executed in accordance with the Declaration of Helsinki and received approval from the Institutional Review Board of the Union Hospital of Fujian Medical University (2023KY042). Informed consent was acquired from all individuals before their inclusion in the study. BM-MSCs were extracted from 30 patients with MPN and 5 healthy donors (**Supplementary Table 1**). Patients were diagnosed with MPN in accordance with the World Health Organization (WHO) 2022 classification (25).

### Isolation and culture of BM-MSCs

Bone marrow aspirates were collected in EDTA-anticoagulated tubes and diluted with phosphate-buffered saline (PBS). Mononuclear cells were extracted via density gradient centrifugation utilizing human peripheral blood lymphocyte separation media (TBD Scientific, China). After centrifugation, the mononuclear cell layer at the interface was carefully collected, washed twice with PBS, and resuspended in α-MEM supplemented with 20% FBS. Cells were then seeded into culture dishes and incubated at 37 °C in a humidified atmosphere containing 5% CO_2_. After 48 h, non-adherent cells were eliminated by media replacement, and the adherent cells were then grown and expanded. The culture media was altered every 2–3 days until the cells attained approximately 80% confluence.

### PBMC isolation

Approximately 3–5 mL of peripheral blood was collected from healthy donors and patients with MPN in EDTA-anticoagulated tubes. PBMCs were isolated by density gradient centrifugation using human peripheral blood lymphocyte separation medium (TBD Scientific, China) according to the manufacturer’s instructions. After centrifugation, the mononuclear cell layer at the plasma–medium interface was collected, washed twice with PBS, and resuspended in RPMI-1640 medium supplemented with 10% FBS for subsequent experiments. Cell viability was assessed by trypan blue exclusion prior to use.

### Bone marrow biopsy and histological analysis

Bone marrow biopsy specimens were collected from patients diagnosed with MPN. The samples were fixed in 10% neutral-buffered formalin, embedded in paraffin, and sectioned at 3–4 μm thickness. Masson staining was performed to evaluate general morphology. Immunohistochemical staining was conducted using antibodies against relevant markers. Sections were visualized under a light microscope, and staining intensity and distribution were independently evaluated by two pathologists.

### Cell cycle, apoptosis, and proliferation assays

Cell viability was assessed using the CCK-8 assay (Yeasen, Shanghai, China), and absorbance at 450 nm was recorded with microplate reader. Cell apoptosis was evaluated by flow cytometry following double staining with Annexin V (BioLegend, San Diego, USA) and 7-AAD (BioLegend, San Diego, USA). For cell cycle analysis, cells were pre-fixed in 75% ethanol at −20 °C overnight and subsequently stained with propidium iodide (PI; BD, USA) before flow cytometric detection.

### Flow cytometry analysis of mesenchymal stromal cell surface markers

For surface staining, the following antibodies were used: APC anti-human CD90 (clone 5E10), PE anti-human CD105 (clone SN6h), FITC anti-human CD73 (clone AD2), and Brilliant Violet 421™ anti-human CD45 (clone 2D1). Cell viability was assessed using the Zombie Aqua™ Fixable Viability Kit, which was detected in the BV510 channel. Stained cells were acquired on a BD FACS Celesta flow cytometer, and data were analyzed using FlowJo software (**Supplementary Table 2**).

### Hydroxyproline assay

Hydroxyproline (HYP) levels in BM-MSCs-conditioned medium were measured using a Hydroxyproline Assay Kit (Yeasen, Cat#60432ES50) according to the manufacturer’s instructions. Following the manufacturer’s instructions, we collected the culture supernatants and spun them to get rid of any debris. Samples (1 mL) were mixed with an equal volume of alkaline hydrolysis solution and incubated at 95 °C for 20 min. After cooling, the pH was set to 6.0–6.8, and distilled water was added to the hydrolysate to make it 10 mL. Following activated carbon clarification, 1 mL of it was mixed with the provided colorimetric reagents (Reagents 1, 2, and 3) in sequence. The reaction mixture was then incubated at 60 °C for 15 minutes. Absorbance was measured at 550 nm, and HYP concentrations were calculated using a standard provided by the kit.

### Immunofluorescence

Cells were fixed in 4% paraformaldehyde (Servicebio, China) for 15 min, rinsed three times with PBS (5 min each), and permeabilized with 0.1% Triton X-100 (Beyotime, China) for 15 min at room temperature. After blocking with 2% FBS for 1 h, the samples were incubated overnight at 4 °C with primary antibodies. Subsequently, cells were exposed to Alexa Fluor^®^ 488- or 555-conjugated anti-rabbit IgG secondary antibodies (ABclonal, China) at room temperature. Nuclei were counterstained with DAPI (Servicebio, China) for 10 min in the dark, and images were acquired using a Leica fluorescence microscope (Leica, Germany).

### Transmission electron microscopy

Cells were inoculated into 10 cm plates and treated with 100 nmol/L ABT263 (or vehicle control) for 24 h. Cell pellets were then collected from both groups, fixed with 2.5% glutaraldehyde for 24 h, and washed three times with PBS. After that, the cells were post-fixed with 1% osmium tetroxide in 0.1 M PBS for 1.5 hours at room temperature. After dehydration in a graded series of acetone, the cells were embedded in pure EMBed 812 and cut into 70-nm-thick ultrathin sections. A transmission electron microscope (HITACHI, Japan) was used to examine the samples after they had been stained with uranyl acetate and lead citrate.

### Reagents

All small-molecule inhibitors were purchased from commercial suppliers: ABT-263(Navitoclax; Selleck Chemicals, USA), and ruxolitinib (Selleck Chemicals, USA). Compounds were prepared following the manufacturer’s instructions, and purity (>98%) was confirmed before experimental application. Recombinant human TGF-β1 was purchased from Proteintech, with a reported purity of >95%.

### Western blotting

Total cellular proteins were extracted by lysing cells in RIPA buffer (Beyotime, Wuhan, China) for 30 minutes, followed by ultrasonic disruption. Immunoblotting was conducted following the usual Bio-Rad technique. After a blocking technique using 5% non-fat milk, nitrocellulose membranes were incubated with specified primary antibodies and corresponding secondary antibodies (**Supplementary Table 3**). The primary antibodies were detected utilizing an enhanced chemiluminescence reagent (Cat. #32106, Thermo Fisher Scientific, Waltham, USA) and imaged with a ChemiDoc Imaging System (Bio-Rad, USA).

### Data acquisition and processing

The Oregon Health and Science University (OHSU) Beat AML dataset: RNA sequencing data (RPKM) and clinical information of AML patients were obtained from the cBioPortal data portal (https://www.cbioportal.org/) (26). The GEO dataset GSE103237: RNA sequencing data (FPKM) and corresponding clinical annotations were downloaded from the Gene Expression Omnibus (GEO) database (https://www.ncbi.nlm.nih.gov/geo/). We analyzed genome-wide CRISPR screen data along with RNA sequencing (RNA-seq) data from AML cell lines available in the DepMap portal.(https://depmap.org/portal/download/all/).

### Mouse xenograft experiments

For studies evaluating the *in vivo* efficacy of ABT-263 (navitoclax), cell line–derived xenograft (CDX) models were established as follows. Briefly, 4*10^6^ luciferase-expressing SET-2 cells were intravenously injected into NCG (NOD-Prkdc^em26Cd52 Il2rg^em26Cd22/NjuCrl) mice. Leukemia engraftment was verified on day 7 after injection through bioluminescence imaging (BLI) with the Vivo Imaging System (Spectral Instruments Imaging). The mice were then randomly assigned to different treatment groups and received either ABT-263 (50 mg/kg, oral gavage, once daily) or vehicle control from day 7 to day 28. Disease progression was tracked on day 21 using BLI. A separate cohort of mice was created following the same protocol for assessing the pharmacodynamics of BCL-XL inhibition *in vivo*.

### Statistics

Statistical analyses were conducted utilizing GraphPad Prism (version 10.0). Results were presented as the mean ± standard deviation (SD) obtained from at least three independent experiments. Differences between the two groups were assessed using either paired or unpaired two-sided Student’s t-tests, contingent upon the experimental design. Comparisons among many groups were assessed using standard one-way analysis of variance (ANOVA). Statistical significance was established as a p-value inferior to 0.05.

## Results

### BCL-XL is overexpressed in MPN progression

**Figure 1:**
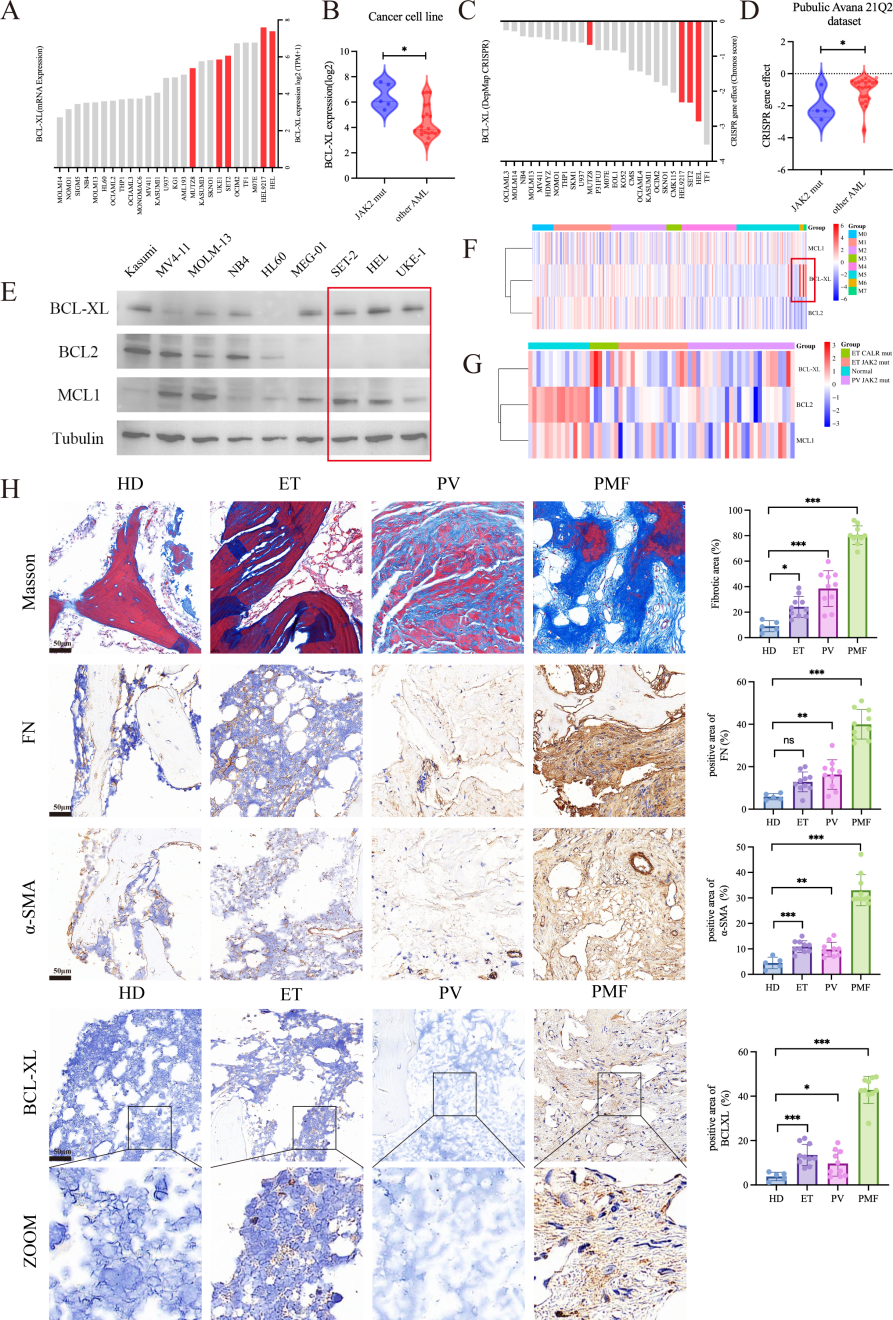
BCL-XL is overexpressed during MPN progression and correlates with bone marrow fibrosis. **(A)** Analysis of the Cancer Cell Line Encyclopedia revealed BCL-XL mRNA expression across AML cell lines. **(B)** Stratification of these AML cell lines according to JAK2 mutational status demonstrated significantly higher BCL-XL mRNA expression in JAK2-mutant lines compared with other AML cell lines (P < 0.05). **(C)** Dependency of AML cell lines on BCL-XL was evaluated using CRISPR knockout screening data from the Public Avana 21Q2 (DepMap) dataset, in which more negative gene effect scores reflect increased cellular dependence. **(D)** Consistent with expression data, JAK2-mutant AML cell lines exhibited significantly lower BCL-XL CRISPR gene effect scores than other AML cell lines, indicating a stronger functional reliance on BCL-XL (P < 0.05). **(E)** Western blot analysis showing the expression of anti-apoptotic BCL-2 family proteins (BCL-XL, BCL2, and MCL1) across a panel of human leukemia cell lines (Kasumi, MV4-11, MOLM-13, NB4, HL-60, MEG-01, SET-2, HEL, and UKE-1). Tubulin served as the loading control. Higher BCL-XL expression was observed in JAK2-mutated MPN-derived cell lines (SET-2, HEL, and UKE-1) compared with other leukemia cell lines. **(F)** Analysis of the OHSU dataset showed that BCLXL expression was markedly elevated in acute myeloid leukemia subtypes M6 and M7, suggesting an association with erythroid and megakaryoblastic leukemias. **(G)** Expression of BCL-XL in CD34+ cells from bone marrow of patients with MPN carrying JAK2V617F ET (n = 17), JAK2V617F PV (n = 26), or CALR-mutant ET (n = 7), as well as from unaffected individuals (n = 15), was analyzed by microarray. Each dot represents one patient. **(H)** Masson staining and immunocytochemistry assays for FN, α-SMA, and BCL-XL were performed on bone marrow samples from healthy donors (HDs) (n = 5) and from patients diagnosed with PV (n = 10), ET (n = 10), and PMF (n = 10). Scale bar = 50 μm. Data are presented as the mean ± SD. (*P<0.05, **P<0.01, ***P<0.001).

To explore the role of anti-apoptotic BCL-2 family members in myeloid malignancies, interrogation of the Cancer Cell Line Encyclopedia demonstrated that BCL-XL expression was markedly elevated in JAK2-mut AML cell lines relative to other AML cell lines (**Figures 1A, B**). Furthermore, analysis of CRISPR screening data from the Public Avana 21Q2 dataset revealed that JAK2-mut cell lines exhibited more negative BCL-XL gene effect scores than other AML cell lines, indicating a stronger functional dependence on BCL-XL (**Figures 1C, D**).

We profiled nine human leukemia cell lines and observed that BCL-XL expression was selectively high in JAK2-mutant HEL, SET-2, and UKE-1 cells. Conversely, BCL-2 and MCL-1 levels were comparatively diminished in these cell lines, suggesting a greater reliance on BCL-XL for cellular viability in the context of JAK2 mutations (**Figure 1E**). Subsequently, we examined BCL-XL expression utilizing the OHSU dataset, we found that BCL-XL was significantly increased in acute myeloid leukemia subtypes M6 and M7, indicating a possible correlation with erythroid and megakaryoblastic leukemias (**Figure 1F**). Analysis of the GEO dataset GSE103237 consistently demonstrated obvious elevated BCL-XL expression in patient samples of PV and ET relative to healthy donors (**Figure 1G**). These data collectively underscored BCL-XL as a pivotal survival factor in JAK2-driven myeloproliferative neoplasms.

To assess bone marrow fibrosis (BMF), paraffin-embedded bone marrow slices from patients with PV, ET, and PMF, as well as from healthy donors (HD), were subjected to Masson staining, in which collagen fibers appear blue. As expected, samples from PMF patients exhibited thick, centrally linked reticulin and collagen fibers extensively disseminated throughout the marrow (**Figure 1H**). In contrast, ET specimens demonstrated modest perivascular collagen deposition, while PV samples displayed an intermediate level of collagen accumulation. Additionally, immunohistochemical analysis of human bone marrow tissues showed significantly higher BCL-XL expression, accompanied by elevated fibrosis markers of α-SMA and FN, especially in PMF samples.

### BM-MSCs from MPN patients exhibit a profibrotic phenotype

**Figure 2:**
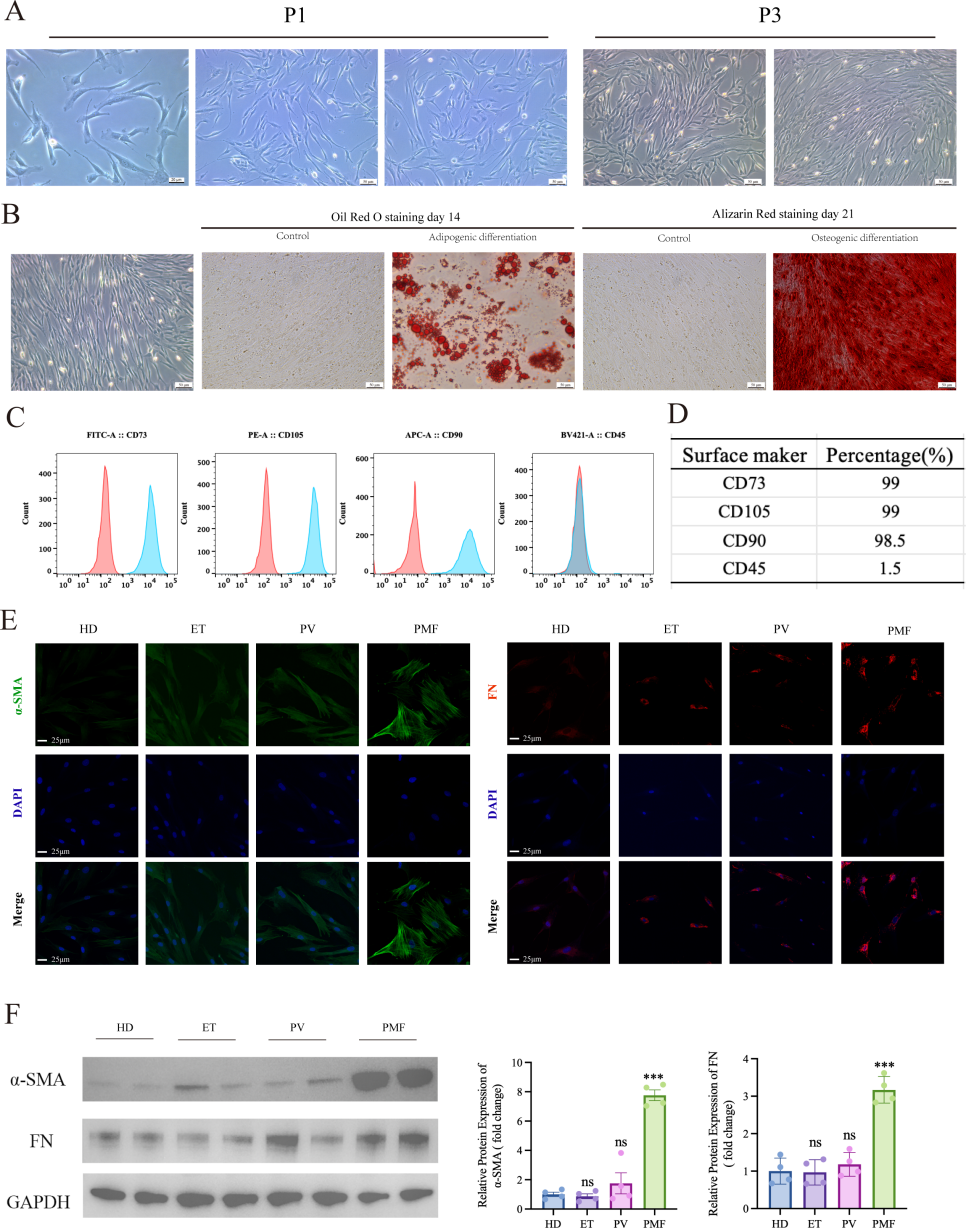
Mesenchymal stromal cells derived from the bone marrow of MPN patients exhibit fibrotic features. BM-MSCs were obtained from HDs and patients diagnosed with PV, ET, or PMF. **(A, B)** Undifferentiated BM-MSCs exhibited fibroblast-like morphology and were capable of lineage differentiation. After 14 days of adipogenic induction, lipid droplets were visualized by Oil Red O staining, whereas osteogenic differentiation after 21 days was confirmed by Alizarin Red staining (n = 3). Scale bar = 50 μm. **(C, D)** Flow cytometric analysis showing the characteristic surface antigen profile of BM-MSCs. Representative gating plots (left) and quantification (right) demonstrate expression of CD73, CD90, and CD105, with negative staining for CD45. **(E)** Immunofluorescence staining of BM-MSCs from HDs and patients with PV, ET, and PMF. FN and α-SMA are shown in red and green, and nuclei are counterstained with DAPI (blue) (n = 3). Scale bar = 25 μm. **(F)** Western blot analysis showing the protein expression levels of FN and α-SMA in BM-MSCs. Quantitative analysis of the western blot results in FN and α-SMA proteins (n = 4). GAPDH was used as the loading control. Data are shown as fold change relative to the control group after normalization to GAPDH. Data are presented as the mean ± SD. (*P<0.05, **P<0.01, ***P<0.001).

To characterize the contribution of BM-MSCs to MPN, we isolated BM-MSCs from both healthy donors and MPN patients. Extracted BM-MSCs appeared short and spindle-shaped with poor adherence when observed under an optical microscope at the early stage of primary culture. After passaging to the first generation (P1), the cells exhibited extended pseudopodia, a loose arrangement, and enlarged cell bodies. When subcultured to the third passage (P3), cell density reached approximately 80%, with good growth status, and the cells showed marked morphological differentiation, displaying a typical elongated spindle-shaped morphology (**Figure 2A**). Morphologically, the cultured cells displayed a stellate, fibroblast-like appearance, and retained the capacity to differentiate into adipocytes and osteoblasts under lineage-specific conditions (**Figure 2B**). As BM-MSCs have been shown to constitute a predominant cellular source responsible for the development of BMF (27, 28). Flow cytometric analysis was used to confirm the identity of BM-MSCs, showing robust expression of the stromal markers CD73, CD90, and CD105, with negative staining of the hematopoietic marker CD45 (**Figures 2C, D**). We next evaluated fibrotic features in BM-MSCs. Immunofluorescence staining and Western blot analyses demonstrated that BM-MSCs derived from MPN patients exhibited significantly increased α-SMA positivity and elevated expression of FN compared with BM-MSCs from healthy controls (**Figures 2E, F**). These findings indicate that BM-MSCs in MPN acquire a myofibrotic phenotype characterized by enhanced expression of profibrotic markers.

### Stromal cells in primary myelofibrosis exhibit elevated BCL-XL expression and enhanced sensitivity to BCL-XL inhibition

**Figure 3:**
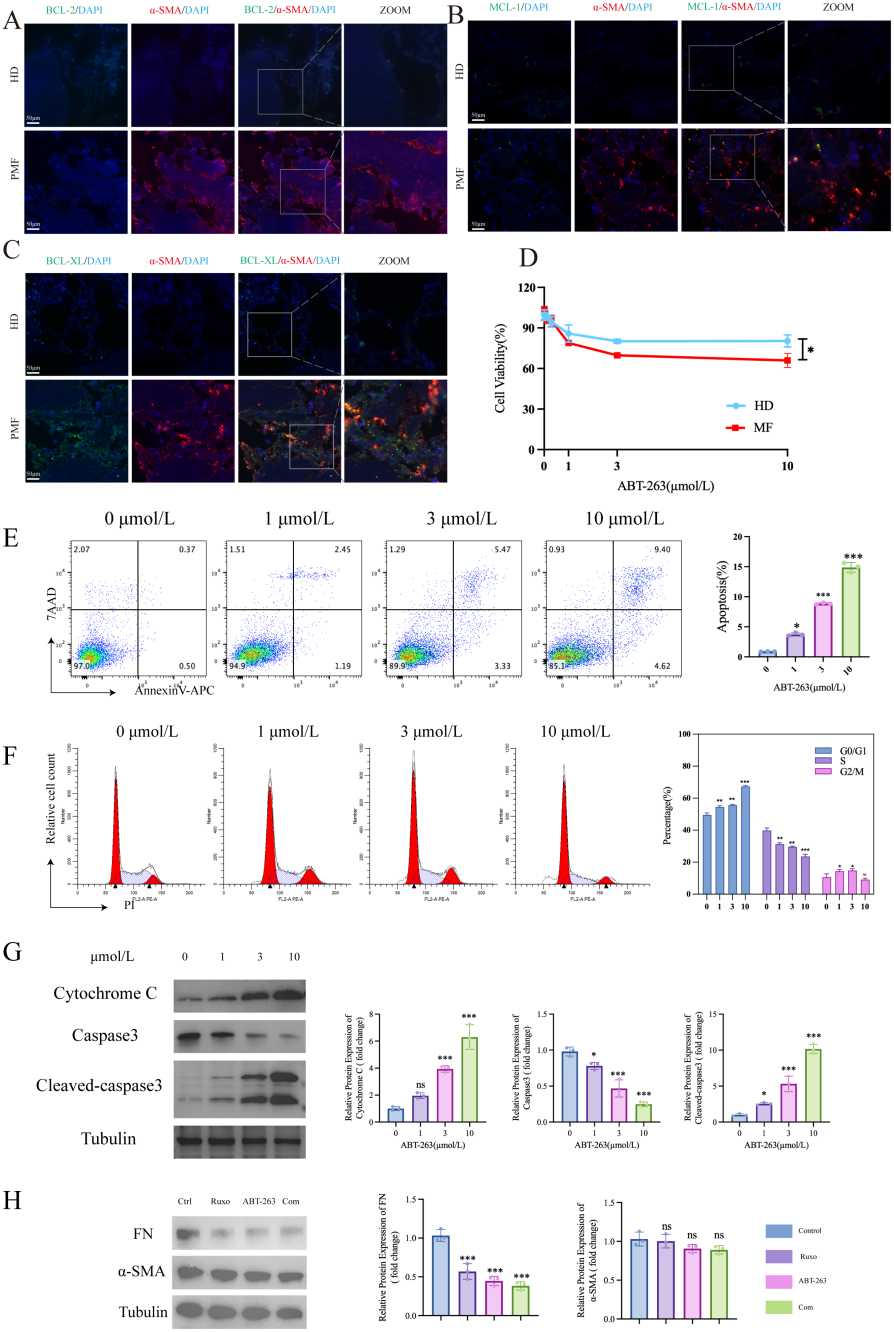
ABT-263 promotes apoptosis and cell-cycle arrest in PMF-MSCs. **(A–C)** α-SMA+ fibrotic fibroblasts express anti-apoptotic BCL-2 family members in bone marrow fibrosis. Immunofluorescence imaging of bone marrow from healthy donors and patients with bone marrow fibrosis was performed for anti-α-SMA (red), DAPI (blue), and anti-apoptotic BCL-2 family members (green), including BCL-2, MCL-1, and BCL-XL. (n = 4). Scale bar = 25 μm. **(D)** CCK-8 assay showing cell viability of BM-MSCs derived from HD and PMF patients after treatment with the indicated concentrations (0, 0.01, 0.03, 0.1, 0.3, 1, 3, and 10 µmol/L) of ABT-263 for 48 h (n = 3). **(E, F)** Flow cytometric analysis of apoptosis and cell cycle distribution in PMF-MSCs after 48 h treatment with ABT-263 at the indicated concentrations (0, 1, 3, and 10 µmol/L) (n = 3). **(G)** Western blot analysis of cytochrome c, caspase-3, and cleaved caspase-3 in PMF-MSCs treated with ABT-263 at the indicated concentrations (0, 1, 3, and 10 µmol/L) for 48 h. Tubulin was used as an internal control (n = 3). Data are shown as fold change relative to the control group after normalization to Tubulin. **(H)** PMF-MSCs treated with ABT-263 (1 µmol/L) and Ruxolitinib (2 µmol/L) for 72 h. Western blot was used to measure the expression of FN and α-SMA proteins. GAPDH was used as the loading control (n = 3). Data are shown as fold change relative to the control group after normalization to GAPDH. Ctrl: Control, Ruxo: Ruxolitinib, Com: combination group. Data are presented as the mean ± SD. (*P<0.05, **P<0.01, ***P<0.001).

Since fibroblasts are recognized as key profibrotic effector cells in tissue fibrosis, we next investigated whether these cells express anti-apoptotic BCL family members in bone marrow samples from patients with MF and healthy donors. Co-immunofluorescence staining revealed that stromal cells in MF marrow exhibited strong co-localization with BCL-XL, whereas BCL-2 and MCL-1 expression was minimal in these regions (**Figures 3A–C**). This pattern indicates that BCL-XL, rather than BCL-2 or MCL-1, is the predominant survival factor within the fibrotic microenvironment of PMF. These findings suggest that BCL-XL plays a crucial role in maintaining the survival of fibrotic stromal cells within the PMF bone marrow niche. Given that PMF-derived MSCs exhibit activated myofibroblast-like features and contribute to excessive extracellular matrix (ECM) deposition, the selective induction of apoptosis by ABT-263 in these cells may represent a potential antifibrotic mechanism.

To functionally validate this finding, bone marrow samples from PMF patients and healthy controls were treated ex vivo with the BCL-XL inhibitor ABT-263. CCK-8 assays demonstrated a pronounced reduction in cell viability in PMF-derived MSCs compared with HD-MSCs following ABT-263 exposure (**Figure 3D**). Flow cytometric analysis revealed that treatment of PMF-MSCs with ABT-263 at concentrations of 1 μM, 3 μM, and 10 μM induced a dose-dependent increase in apoptotic cells (**Figure 3E**). Cell-cycle profiling showed a progressive accumulation of cells in the G0/G1 phase accompanied by a reduction of the S-phase population, indicating growth arrest (**Figure 3F**). Western blot analysis further demonstrated a concentration-dependent elevation of cleaved-caspase3 and cytochrome c expression, whereas total caspase-3 levels decreased (**Figure 3G**), confirming activation of the intrinsic mitochondrial apoptotic pathway. The enhanced release of cleaved-caspase 3 and cytochrome c observed in PMF-MSCs indicates activation of the intrinsic mitochondrial apoptotic pathway, which may facilitate the elimination of myofibroblastic stromal cells with high expression of α-SMA and FN. Consequently, targeting BCL-XL not only disrupts the survival advantage of PMF-MSCs but may also attenuate the fibrogenic remodeling of the marrow microenvironment, thereby restoring a more physiologic hematopoietic niche.

To evaluate the antifibrotic effect of BCL-XL targeting via modulation of the profibrotic stromal cell phenotype, PMF-MSCs were treated with Ruxolitinib alone or in combination with ABT-263. Compared with Ruxolitinib monotherapy, the combination treatment resulted in a more pronounced reduction of FN and α-SMA expression (**Figure 3H**), indicating a synergistic antifibrotic effect. This cooperation likely arises from dual inhibition of pro-survival and cytokine-driven pathways: while Ruxolitinib suppresses JAK-STAT–mediated inflammatory signaling, ABT-263 eliminates apoptosis-resistant stromal cells by blocking BCL-XL.

### MPN mononuclear cells drive fibrotic activation of BM-MSCs

To investigate whether malignant hematopoietic cells contribute to stromal activation, we performed co-culture experiments using healthy donor–derived BM-MSCs and PBMCs obtained from healthy donors or patients with JAK2-mutant MPN, including ET, PV, and PMF.

Compared with HD BM-MSCs cultured alone or co-cultured with PBMCs from healthy donors, co-culture with PBMCs from patients with ET, PV, or PMF for 48 h resulted in increased FN and α-SMA expression in BM-MSCs, as assessed by immunofluorescence (**Supplementary Figure 1**). These results indicate that MPN-derived mononuclear cells facilitate BM-MSC activation via microenvironmental interactions.

To further investigate the signaling mechanisms underlying MSC activation, we next examined the TGF-β/SMAD3 and JAK2/STAT3 pathways.

### Activation of TGF-β/SMAD3 and JAK2/STAT3 signaling in MPN-derived mesenchymal stromal cells and effects of pathway inhibition

**Figure 4:**
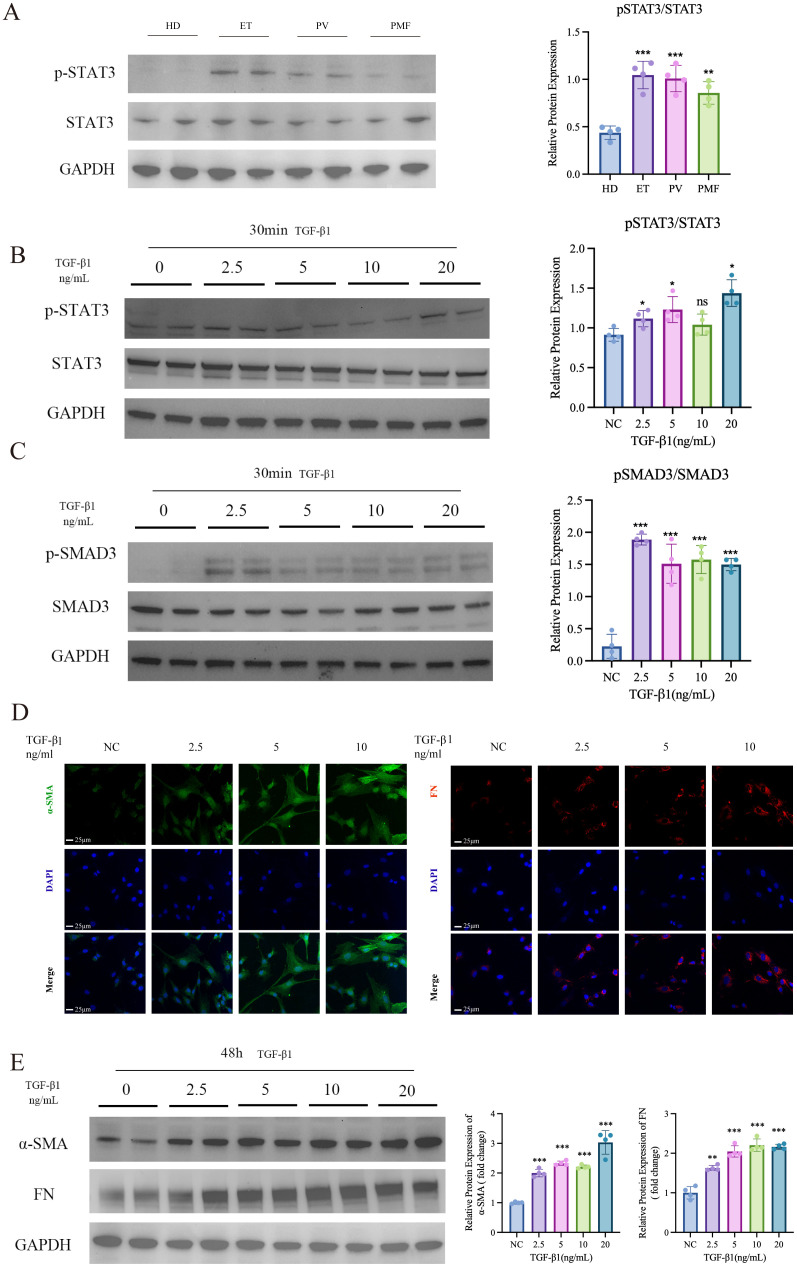
TGFβ/SMAD3 and JAK2/STAT3 signaling in bone marrow mesenchymal stromal cells derived from MPN patients. **(A, B)** The Western blot analysis revealed the expression of p-STAT3, and total STAT3 in BM-MSCs from HDs and patients with PV, ET, and PMF. GAPDH and tubulin were used as internal controls (n = 3). **(C, D)** HD BM-MSCs were treated with the specified concentrations of TGF-β1 for 30 minutes. Western blot analysis shows the protein expression levels of STAT3, pSTAT3, SMAD3, and pSMAD3. Quantitative analysis of the western blot results in these proteins. GAPDH was used as an internal control (n = 3). **(E)** HD BM-MSCs were treated with the specified concentrations of TGF-β1 for 48 h. We used immunofluorescence staining and performed a western blot of FN and -SMA. FN and α-SMA are shown in red and green, and nuclei are counterstained with DAPI (blue). Quantitative analysis of the western blot results in FN and α-SMA proteins. GAPDH was used as the loading control (n = 4). Data are shown as fold change relative to the control group after normalization to GAPDH. Scale bar = 25 μm. Data are presented as the mean ± SD. (*P<0.05, **P<0.01, ***P<0.001).

To characterize the profibrotic signaling networks in MPN–derived BM-MSCs, we examined the expression of key mediators of the TGF-β/SMAD3 and JAK2/STAT3 pathways. Phosphorylated STAT3 (pSTAT3) was markedly elevated in MPN-MSCs relative to HD controls (**Figure 4A**), consistent with constitutive activation of the JAK/STAT pathway within the fibrotic microenvironment.

To further confirm the interaction between TGF-β1 and downstream signaling cascades, healthy BM-MSCs were stimulated with recombinant TGF-β1 for 30 minutes (**Figures 4B, C**). TGF-β1 exposure led to a dose-dependent increase in phosphorylated SMAD3 (pSMAD3) and pSTAT3, demonstrating functional convergence of the TGF-β/SMAD3 and JAK2/STAT3 pathways.

We next assessed whether TGF-β signaling directly promotes a fibrotic phenotype in BM-MSCs (**Figures 4D, E**). Healthy donor–derived MSCs were treated with graded concentrations of TGF-β1 (0 ng/mL, 2.5 ng/mL, 5 ng/mL, 10 ng/mL, 20 ng/mL) for 48 h. Immunofluorescence staining revealed a progressive accumulation of FN and α-SMA in response to TGF-β1, with enhanced FN network formation and cytoplasmic α-SMA filament assembly. Western blot analysis further confirmed a concentration-dependent upregulation of FN and α-SMA protein levels, consistent with myofibroblastic differentiation and extracellular matrix synthesis. Quantitative analysis demonstrated significant increases at TGF-β1 concentrations as low as 2.5 ng/mL.

To further investigate the functional role of the TGF-β/SMAD3 pathway in MSC fibrotic activation, fibrotic MSCs were treated with galunisertib (LY2157299), a potent and selective TGF-β receptor I (TGF-βRI/ALK5) inhibitor that specifically abrogates the canonical TGF-β/SMAD pathway via inhibition of SMAD phosphorylation. Galunisertib was used at 10 µM for 48 h, consistent with concentrations reported in previous *in vitro* studies (29, 30). Treatment of TGF-β1–stimulated MSCs with galunisertib not only suppressed SMAD3 phosphorylation but also attenuated STAT3 activation (**Supplementary Figure 2A**). Galunisertib also reduced FN and α-SMA expression in MSCs induced by TGF-β1 (**Supplementary Figure 2B**).

Collectively, these findings demonstrate that TGF-β1 stimulation induces SMAD3 and STAT3 phosphorylation in BM-MSCs, leading to activation of a fibrogenic program characterized by elevated FN and α-SMA expression. This supports a model in which TGF-β/SMAD3 and JAK2/STAT3 signaling cooperate to drive stromal activation and fibrosis in the MPN bone marrow niche.

### Combined inhibition of JAK2 and BCL-XL synergistically reduces TGFβ-induced fibrosis in MSCs

**Figure 5:**
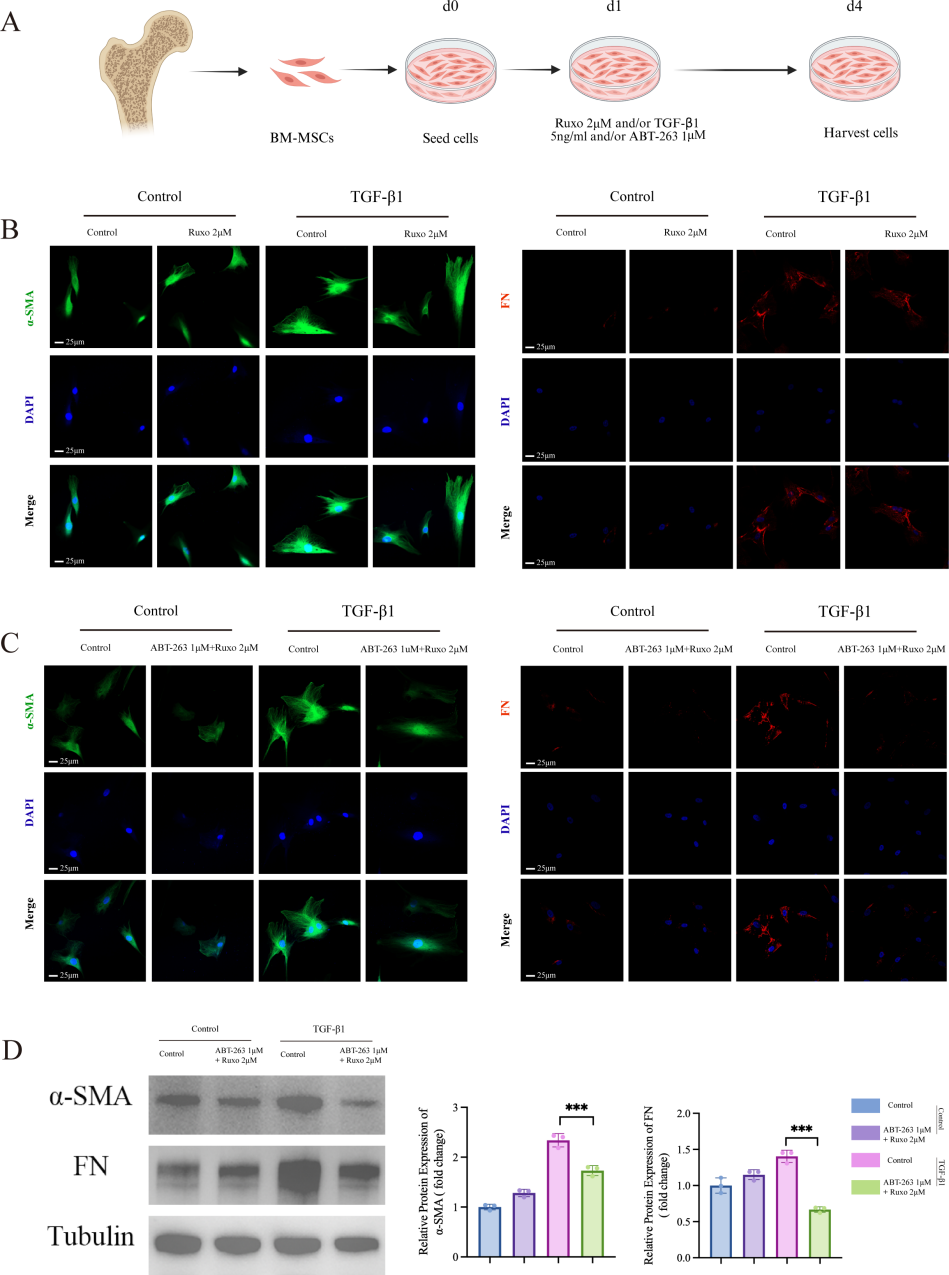
Treatment with ABT-263 and ruxolitinib reduces TGFβ-induced fibrosis in mesenchymal stromal cells. **(A)** Flow chart of bone marrow mesenchymal stromal cells from healthy donors (HD BM-MSCs) were subjected to treatment with transforming TGF-β1 5ng/mL, ruxolitinib 2 μM, ABT-263 1 μM, or a combination thereof. **(B–D)** Immunofluorescence staining and western blot of FN and α-SMA. FN and α-SMA are shown in red and green, and nuclei are counterstained with DAPI (blue). Quantitative analysis of the western blot results in FN and α-SMA proteins. GAPDH was used as the loading control (n = 3). Data are shown as fold change relative to the control group after normalization to GAPDH. Scale bar = 25 μm. Ctrl: Control, Ruxo: Ruxolitinib, Data are presented as the mean ± SD. (*P<0.05, **P<0.01, ***P<0.001).

To determine whether BCL-XL inhibition enhances the antifibrotic effects of JAK2 blockade, HD BM-MSCs were exposed to TGF-β1 for 3 days and subsequently treated with Ruxolitinib, ABT-263, or their combination (**Figure 5A**). Based on preliminary CCK-8 dose-response assays in HD BM-MSCs, we selected 1 μM ABT-263 and 2 μM ruxolitinib for subsequent experiments to effectively modulate fibrotic signaling without inducing significant cytotoxicity (**Supplementary Figures 3A, B**).

After 3 days of stimulation, immunofluorescence staining showed that ruxolitinib alone only slightly reduced α-SMA and FN expression compared with TGF-β1-treated controls, indicating that short-term JAK2 inhibition was insufficient to fully suppress TGF-β1-driven fibrotic activation (**Figure 5B**).

In contrast, combined treatment with ABT-263 and Ruxolitinib led to a marked reduction of both α-SMA and FN expression (**Figures 5C, D**), as demonstrated by immunofluorescence and Western blotting. Consistently, immunofluorescence staining for Collagen I revealed decreased collagen deposition in HD BM-MSCs following dual inhibition, and hydroxyproline assays confirmed a significant reduction in total collagen content (**Supplementary Figures 4A, B**). Dual inhibition reduced α-SMA and FN expression and decreased collagen accumulation induced by TGF-β1. These findings suggest that BCL-XL inhibition potentiates the antifibrotic effect of JAK2 blockade, likely through the elimination of apoptosis-resistant myofibroblast-like MSCs and attenuation of JAK/STAT-dependent profibrotic signaling.

In contrast, MSCs derived from PMF patients, which are already fibrotic *in vivo*, exhibited differential responses: FN expression was inhibited by the combined treatment, whereas α-SMA remained largely unchanged (**Figure 3H**). This observation is consistent with previous reports showing that fibrotic markers exhibit differential reversibility, with extracellular matrix proteins such as FN being more dynamic and cytoskeletal markers such as α-SMA being more resistant once myofibroblast differentiation is established (31–33).

Collectively, these results demonstrate that simultaneous targeting of BCL-XL and JAK2 provides a more effective strategy to mitigate TGF-β1-induced fibrotic remodeling in MSCs than JAK inhibition alone.

### ABT-263 inhibits cell viability and induces apoptosis in post-MPN AML cell lines

**Figure 6:**
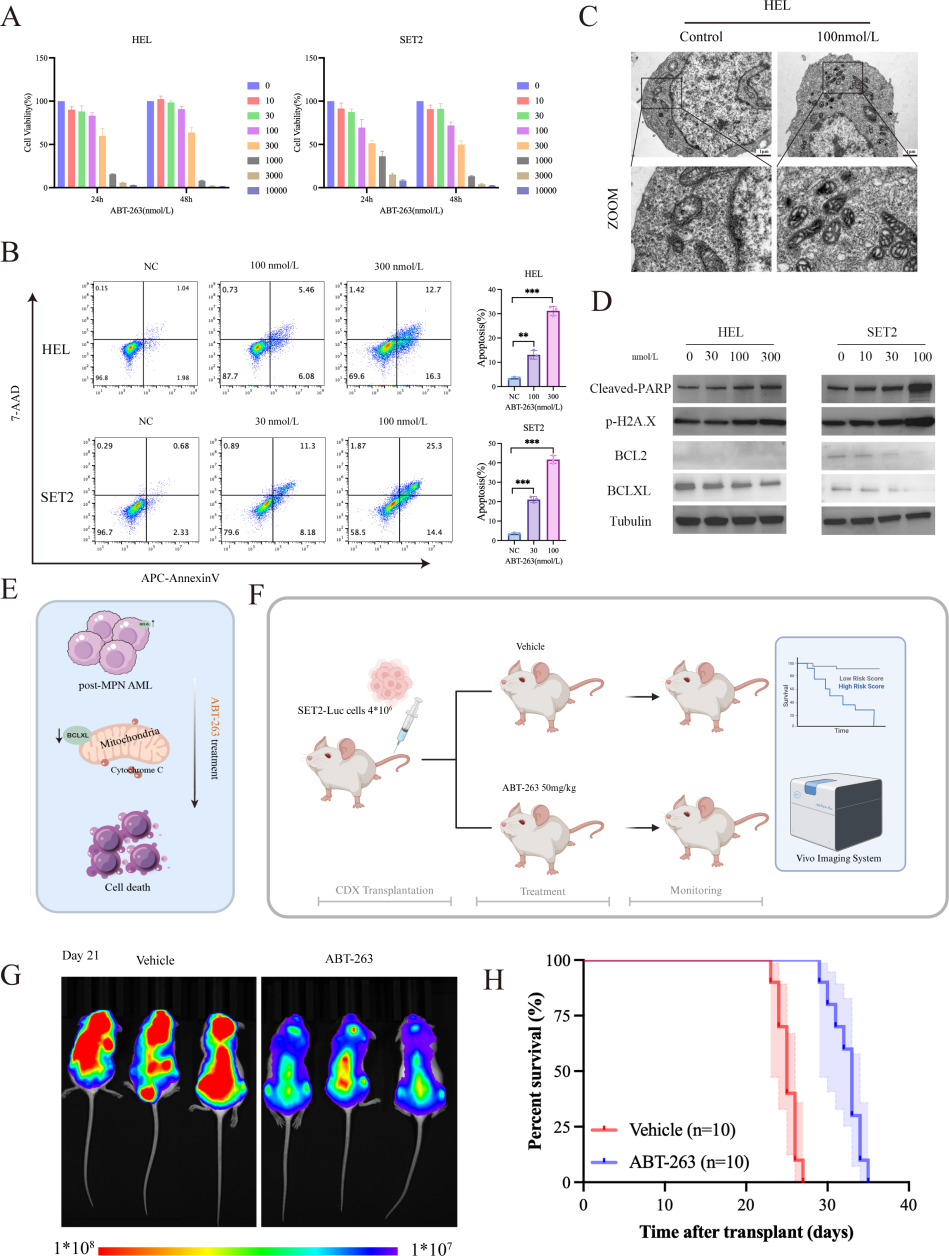
ABT-263 induces mitochondrial apoptosis in JAK2-mutated myeloproliferative neoplasm cell lines. **(A)** CCK8 assay showing dose- and time-dependent reduction in cell viability in HEL and SET2 cells following ABT-263 treatment (0, 10, 30, 100, 300, 1000, 3000, and 10,000 nmol/L, 24–48 h) (n = 3). **(B)** Representative flow cytometry plots of Annexin V–APC/7-AAD staining in HEL and SET2 cells treated with indicated concentrations (0, 100, and 300 nmol/L) of ABT-263 for 24 h (n = 3). Quantitative analysis demonstrates a significant increase in apoptotic populations in a dose-dependent manner. **(C)** Transmission electron microscopy (TEM) images of HEL cells reveal pronounced mitochondrial swelling, disrupted cristae, and vacuolization after exposure to ABT-263 (100 nmol/L) for 24 h, indicative of mitochondrial injury and apoptosis. Scale bar = 1μm. **(D)** Western blot analysis showing increased cleavage of PARP and phosphorylation of H2A.X, accompanied by downregulation of anti-apoptotic BCL-2 and BCL-XL proteins in a concentration-dependent manner (0, 30, 100, and 300 nmol/L). Tubulin served as the loading control (n = 3). Data are shown as fold change relative to the control group after normalization to Tubulin. Data are presented as the mean ± SD. (*P<0.05, **P<0.01, ***P<0.001). **(E)** Schematic diagram of the mechanism by which ABT-263 exerts antitumor effects inpost-MPN AML. **(F)** The process diagram for SET2-luc cells xenograft model construction and ABT-263 intervention therapy. **(G)** Bioluminescence imaging technology to evaluate the anti-leukemia effect of ABT-263 treatment (n = 3). **(H)** The survival of mice treated with the vehicle or ABT-263 (n = 10).

To assess the cytotoxic effect of the BCL-XL inhibitor ABT-263 on post-MPN AML cells, HEL and SET2 cells were exposed to different concentrations of the drug for 24 and 48 h. As shown in **Figures 6A**,ABT-263 treatment led to a dose- and time-dependent decrease in cell viability, with marked suppression observed at concentrations above 300 nmol/L. Flow cytometric analysis confirmed a significant elevation in apoptotic cell populations upon ABT-263 exposure (**Figure 6B**). In both HEL and SET2 cells, the percentage of apoptotic cells increased notably at 100–300 nmol/L compared with vehicle-treated controls (p < 0.001), indicating a robust induction of programmed cell death. Transmission electron microscopy revealed profound ultrastructural alterations following treatment with ABT-263 (100 nmol/L) for 24 h (**Figure 6C**). Treated HEL cells displayed swollen mitochondria with disrupted cristae and condensed chromatin, characteristic of apoptotic morphology, whereas control cells maintained normal organelle integrity. Consistently, Western blot analysis demonstrated increased cleaved-PARP and pH2A.X in ABT-263-treated cells, accompanied by a marked reduction in BCL-XL and BCL-2 protein levels (**Figure 6D**). These results collectively suggest that navitoclax exerts potent pro-apoptotic activity in post-MPN AML cells through inhibition of anti-apoptotic BCL-2 family members and activation of the mitochondrial apoptosis pathway.

Our *in vitro* investigations identified BCL-XL as a viable therapeutic target in post-MPN AML cell lines and patient-derived primary samples (**Figure 6E**). For *in vivo* tracking, SET-2 cells were stably transduced with a luciferase reporter and then injected into NCG mice via the tail vein (**Figure 6F**). The formation of leukemia was confirmed using bioluminescence imaging (BLI), after which subjects were randomly assigned to receive either a vehicle control or ABT-263 for three weeks. Compared to vehicle-treated mice, ABT-263 monotherapy significantly inhibited SET-2 AML disease progression *in vivo*, as indicated by reduced bioluminescent signal intensity (**Figure 6G**) and a notable increase in overall survival (**Figure 6H**).

### Targeting BCL-XL overcomes reduced responsiveness to ruxolitinib in primary PMF patient cells

**Figure 7:**
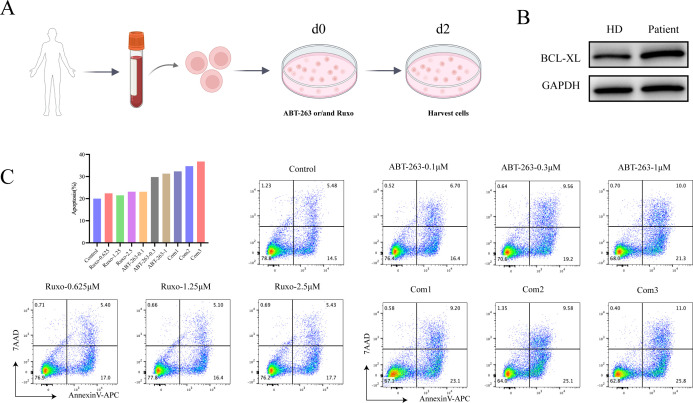
ABT-263 enhances apoptosis in ruxolitinib-resistant PMF PBMCs. **(A)** Schematic diagram of PBMCs from a patient with PMF, treated with ruxolitinib, ABT-263, or their combination. **(B)** Western blot showing BCL-XL protein expression in samples from a healthy donor (HD) and a patient with PMF, with GAPDH as the loading control. **(C)** Nvestigation of drug-induced apoptosis in PBMCs from a 67-year-old primary myelofibrosis patient with the JAK2V617F mutation. PBMCs were treated with varying concentrations of ruxolitinib (0.625 μM, 1.25 μM, and 2.5 μM) for 48 h. Subsequently, PBMCs were treated with ABT-263 at doses of 0.1 μM, 0.3 μM, and 1 μM for 48 h. The combination treatments of ruxolitinib and ABT-263—Com1 (0.625 μM + 0.1 μM), Com2 (1.25 μM + 0.3 μM), and Com3 (2.5 μM + 1 μM)—resulted in significantly higher apoptotic rates compared with either drug alone. Ruxo: Ruxolitinib, Com: combination group.

Peripheral blood mononuclear cells (PBMCs) isolated from a 67-year-old patient with PMF harboring the JAK2V617F mutation, who developed marked leukocytosis (WBC 60.18*10^9^/L) after long-term treatment with ruxolitinib, were subjected to flow cytometric analysis and Western blotting (**Figure 7A**). Western blot analysis showed that BCL-XL expression was markedly elevated in the patient’s peripheral blood cells compared with those from healthy controls (**Figure 7B**). First, PBMCs were treated with ruxolitinib at 0.625 μM, 1.25 μM, and 2.5 μM for 48 h. Across these concentrations, apoptosis rates showed no substantial increase, suggesting that the patient’s leukemic cells exhibited reduced sensitivity or acquired resistance to ruxolitinib. To further explore potential therapeutic vulnerabilities, we next examined the effect of the ABT-263. Treatment with 0.1 μM, 0.3 μM, and 1 μM ABT-263 for 48 h resulted in a dose-dependent increase in apoptosis, indicating that this cell population is susceptible to BCL-XL inhibition. Then, we assessed the effect of combination therapy by pairing each ruxolitinib concentration with a corresponding ABT-263 dose (0.625 μM + 0.1 μM, 1.25 μM + 0.3 μM, and 2.5 μM + 1 μM) (**Figure 7C**). In all conditions, the combination treatment produced higher apoptotic rates than either agent alone, demonstrating a synergistic or additive pro-apoptotic effect. These results imply that BCL-XL pathway inhibition can overcome, at least partially, the reduced responsiveness to JAK inhibition observed in this patient’s cells. Collectively, these findings suggest that while ruxolitinib alone no longer effectively induces apoptosis in this patient’s PBMCs, targeting anti-apoptotic BCL-XL proteins with ABT263—particularly in combination with ruxolitinib—may enhance cell death and provide a potential strategy to circumvent therapeutic resistance.

## Discussion

Bone marrow fibrosis and leukemic transformation pose significant clinical problems in myeloproliferative neoplasms, leading to disease progression, treatment resistance, and unfavorable prognosis. While JAK–STAT signaling is acknowledged as a primary contributor to MPN pathogenesis (15), existing JAK inhibitor medicines do not sufficiently reverse fibrosis or avert leukemic progression, highlighting the necessity to uncover further molecular processes that sustain illness persistence. Our study highlights the essential role of the anti-apoptotic protein BCL-XL as a key survival factor in malignant hematopoietic cells and fibrotic stromal components associated with JAK2-mutant MPN. The findings suggest that targeting BCL-XL represents a rational strategy to overcome apoptosis resistance and mitigate fibrotic remodeling.

Our data indicate that BCL-XL expression is specifically heightened in JAK2-driven MPN and post-MPN AML, while other anti-apoptotic BCL-2 family members, such as BCL-2 and MCL-1, are relatively diminished. This expression pattern was consistently noted across patient datasets, leukemia cell lines, and original samples, demonstrating a preferential reliance on BCL-XL for cellular survival in the JAK2-mutant setting. These findings are consistent with previous studies that JAK–STAT signaling transcriptionally modulates BCL-XL (34), indicating that prolonged JAK2 activation provides a survival benefit by enhancing BCL-XL–mediated mitochondrial protection.

Our research has broadened the understanding of the mechanisms of BCL-XL, extending its influence from malignant hematopoietic cells to the bone marrow stromal environment. BM-MSCs derived from patients with MPN exhibit a distinct myofibroblast-like phenotype, characterized by elevated levels of α-SMA and FN, which is consistent with their established role as key effector cells in bone marrow fibrosis. Our findings indicated that in PMF, BCL-XL expression was significantly elevated, and the BCL-XL inhibitor ABT-263 selectively induces apoptosis in MPN-derived MSCs without affecting MSCs from healthy donors. This suggested that BCL-XL serves as the primary anti-apoptotic protective factor within the fibrotic bone marrow microenvironment, and the selective elimination of myofibroblast-like MSCs with anti-apoptotic capabilities may represent a direct antifibrotic mechanism.

We demonstrate that transforming TGF-β1 induces phosphorylation of both SMAD3 and STAT3 in MSCs, highlighting functional crosstalk between the canonical TGF-β/SMAD and JAK2/STAT3 pathways. The role of STAT3 in TGF-β1 cellular effects during fibrogenesis has already been reported in liver fibrosis, and pulmonary fibrosis (35–38). TGF-β1 stimulation was sufficient to drive FN and α-SMA expression in healthy MSCs, recapitulating key features of the fibrotic phenotype observed in MPN-derived cells. These findings support a model in which inflammatory and profibrotic cues converge on STAT3 activation, reinforcing both fibrotic differentiation and survival signaling. In this context, BCL-XL may function as a critical downstream effector that enables MSCs to withstand pro-apoptotic stress during chronic fibrotic remodeling.

Consistent with this model, combined inhibition of JAK2 and BCL-XL produced synergistic antifibrotic and pro-apoptotic effects. Prior research has demonstrated that JAK2-mutant clones create a persistent inflammatory environment characterized by elevated levels of IL-6, TGF-β1, and PDGF, which promotes the differentiation of myofibroblasts from MSCs and leads to the gradual accumulation of extracellular matrix (21, 39). Ruxolitinib alone had a minimal effect on preexisting fibrotic characteristics, but its combination with ABT-263 significantly reduced α-SMA and FN expression, leading to a restoration of a more quiescent stromal phenotype. This combination presumably indicates simultaneous targeting of upstream inflammatory signaling and downstream survival mechanisms, addressing both the activation and persistence of fibrotic MSCs. In addition to the stromal compartment, ABT-263 exhibited significant pro-apoptotic efficacy in post-MPN AML cell lines and in patient-derived cells with diminished responsiveness to ruxolitinib. The capacity of BCL-XL inhibition to surmount JAK inhibitor resistance highlights its potential translational significance, especially in advanced or refractory disease contexts indicating that BCL-XL constitutes a promising target in both malignant hematopoietic and fibrotic stromal compartments in MPN.

Our observations align with increasing clinical data. Initial trials suggest that ABT-263 in conjunction with ruxolitinib demonstrates favorable disease-modifying effects in myelofibrosis, encompassing reductions in spleen volume, symptom scores, and, crucially, histological enhancement of marrow fibrosis (7). The clinical advantages correspond with our experimental results indicating that simultaneous inhibition of BCL-XL and JAK2 can reverse stromal activation. Thrombocytopenia, however, continues to be a significant constraint of navitoclax because of the physiological reliance of platelets on BCL-XL (40, 41). Our study did not directly assess the platelet count or examine the broader hematopoietic toxicity. Therefore, future research needs to include the monitoring of platelets and hematopoietic function.

A limitation of this study is the lack of genetic validation of BCL-XL, and the xenograft model does not fully recapitulate the MPN-specific bone marrow microenvironment, particularly stromal–hematopoietic interactions and fibrosis development. Although pharmacologic inhibition using ABT-263 supports a functional role for BCL-XL, its dual activity against BCL-2 family members precludes complete specificity. Notably, BCL-2 expression was relatively low compared to BCL-XL in our study, suggesting a predominant role of BCL-XL. Future studies using BCL-XL-targeted genetically engineered MPN mouse models or more selective inhibitors, such as A-1331852, will be important to further validate these findings. It should be noted that PMF is a rare disease, and patients who are resistant to ruxolitinib are even less common, making clinical samples from this population difficult to obtain, which limits the generalizability of our findings. In future studies, more clinical cases of myelofibrosis will enhance the persuasiveness of our research.

In summary, our study identifies BCL-XL as a central mediator of apoptosis resistance in JAK2-mutant MPN, linking malignant cell survival, stromal fibrotic activation, and therapeutic resistance. By demonstrating that combined targeting of BCL-XL and JAK2 effectively disrupts both fibrotic remodeling and leukemic cell survival, our findings provide a rationale for dual-pathway therapeutic strategies aimed at modifying the bone marrow microenvironment and improving outcomes in advanced MPN.

## Data Availability

The original contributions presented in the study are included in the article/**Supplementary Material**. Further inquiries can be directed to the corresponding author.
