# Supplementary material for: BCL-XL drives fibrotic and leukemic progression in myeloproliferative neoplasms
Source: Front Immunol. 2026 Jun 2;17:1818806. doi: 10.3389/fimmu.2026.1818806 (PMC13269428; doi:10.3389/fimmu.2026.1818806)
Supplement: Supplementary file 1 [file DataSheet1.docx]

**
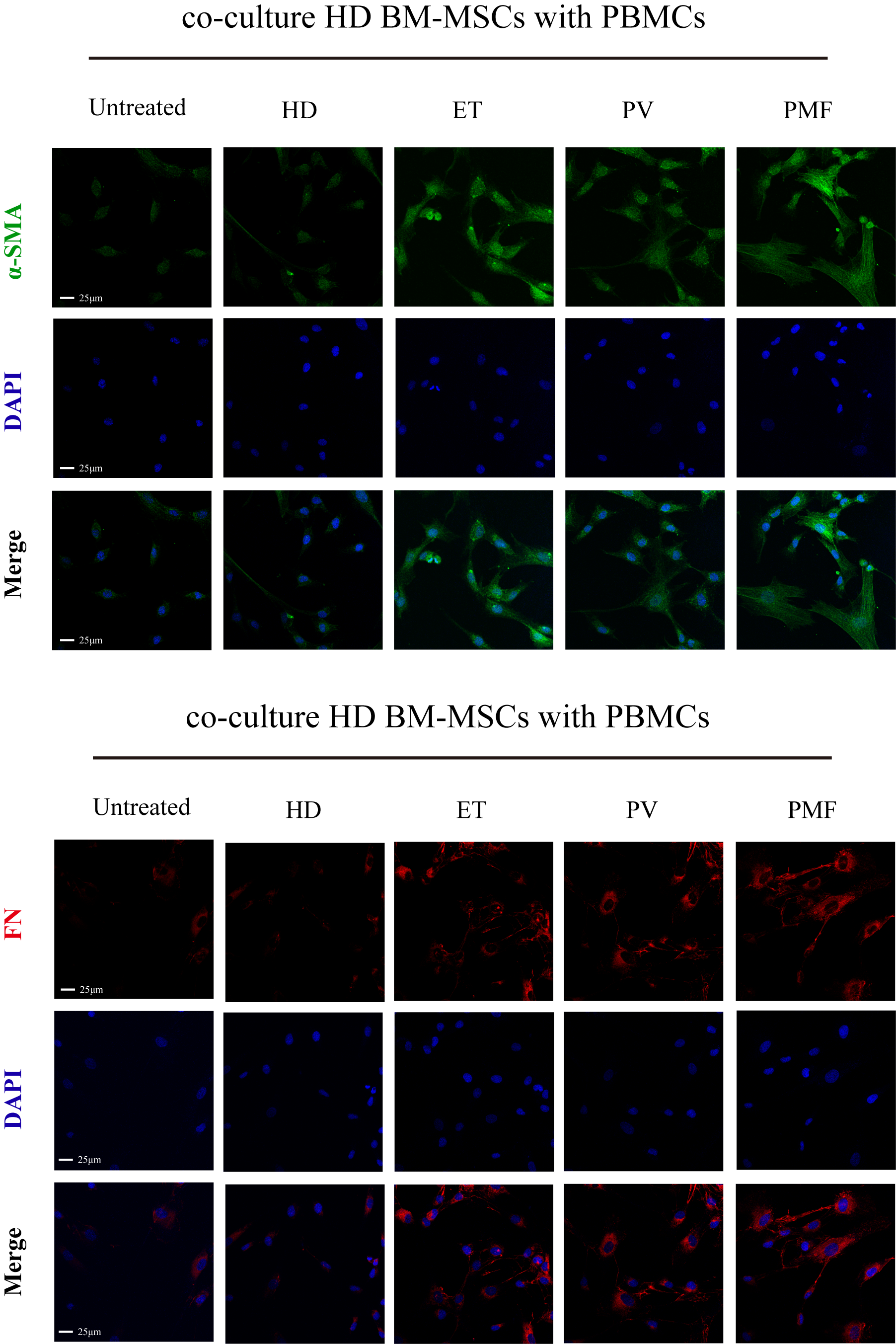
**

**Supplementary Figure 1. MPN mononuclear cells modulate fibrotic responses in BM-MSCs**

Immunofluorescence staining of α-SMA (green) and fibronectin (red) in bone marrow mesenchymal stromal cells from healthy donors (HD BM-MSCs) cultured alone or after 48 h of co-culture with peripheral blood mononuclear cells (PBMCs) from healthy donors or patients with ET, PV, or PMF. Nuclei were counterstained with DAPI (blue). Increased expression of α-SMA and FN was observed in HD BM-MSCs co-cultured with MPN-derived PBMCs compared with monoculture or co-culture with healthy donor PBMCs. healthy donor (HD), polycythemia vera (PV), essential thrombocythemia (ET), and primary myelofibrosis (PMF). Scale bar = 25 μm. n = 3.


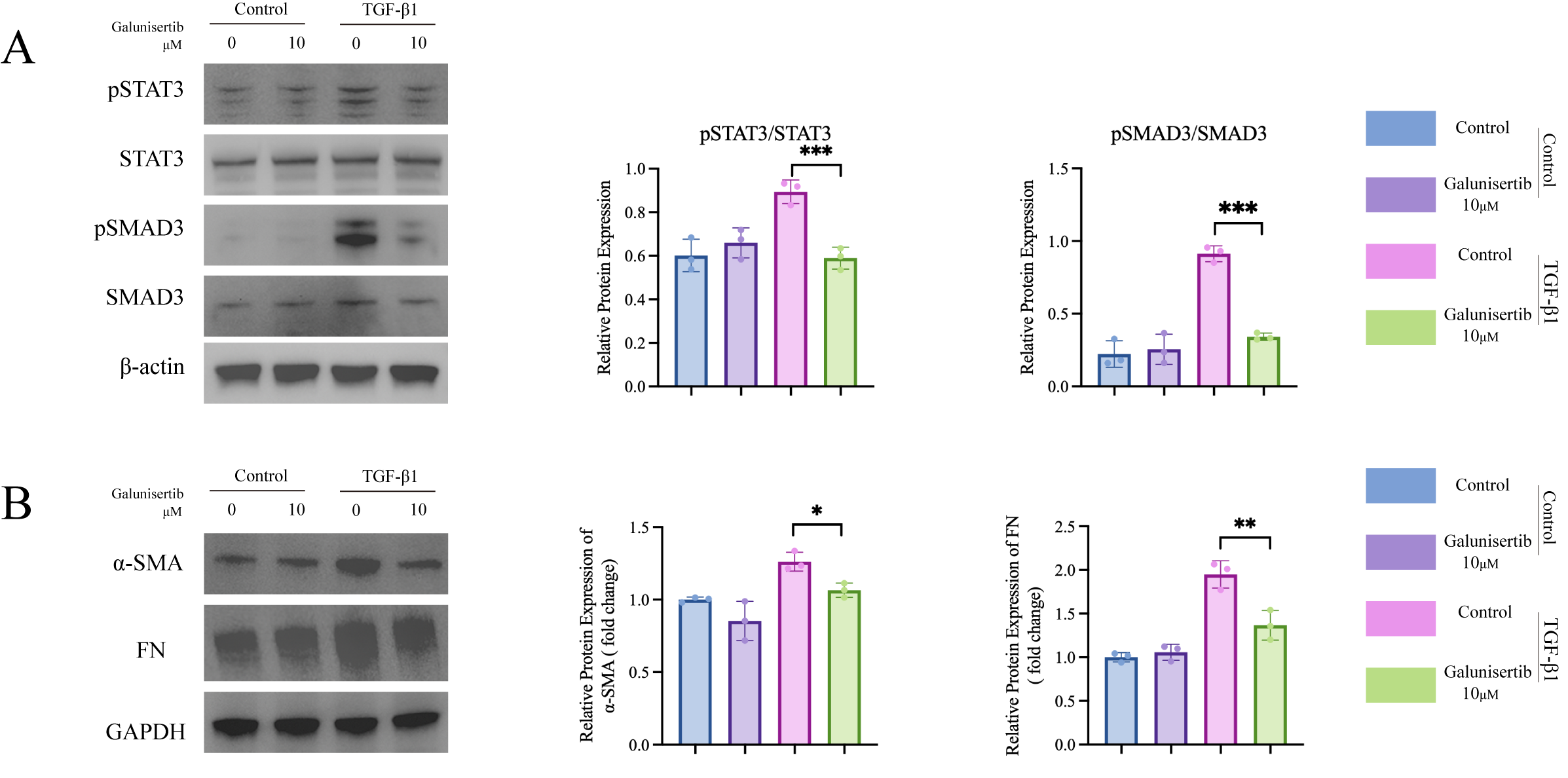


**Supplementary Figure 2. Galunisertib attenuates TGF-β1–induced SMAD3 and STAT3 phosphorylation and reduces fibrotic marker expression in BM-MSCs.**

HD BM‑MSCs were treated with TGF-β1 (5 ng/mL) in the presence of galunisertib (10 μM) for 48 h, and whole‑cell lysates were subjected to western blotting.

(A) Western blot analysis of pSTAT3/STAT3 and pSMAD3/SMAD3 (n = 3).

(B) Western blot analysis of FN and α-SMA (n = 3).

Data are shown as fold change relative to the control group after normalization to β-actin or GAPDH. Data are shown as mean ± SD. *p < 0.05, **p < 0.01,***p < 0.001.


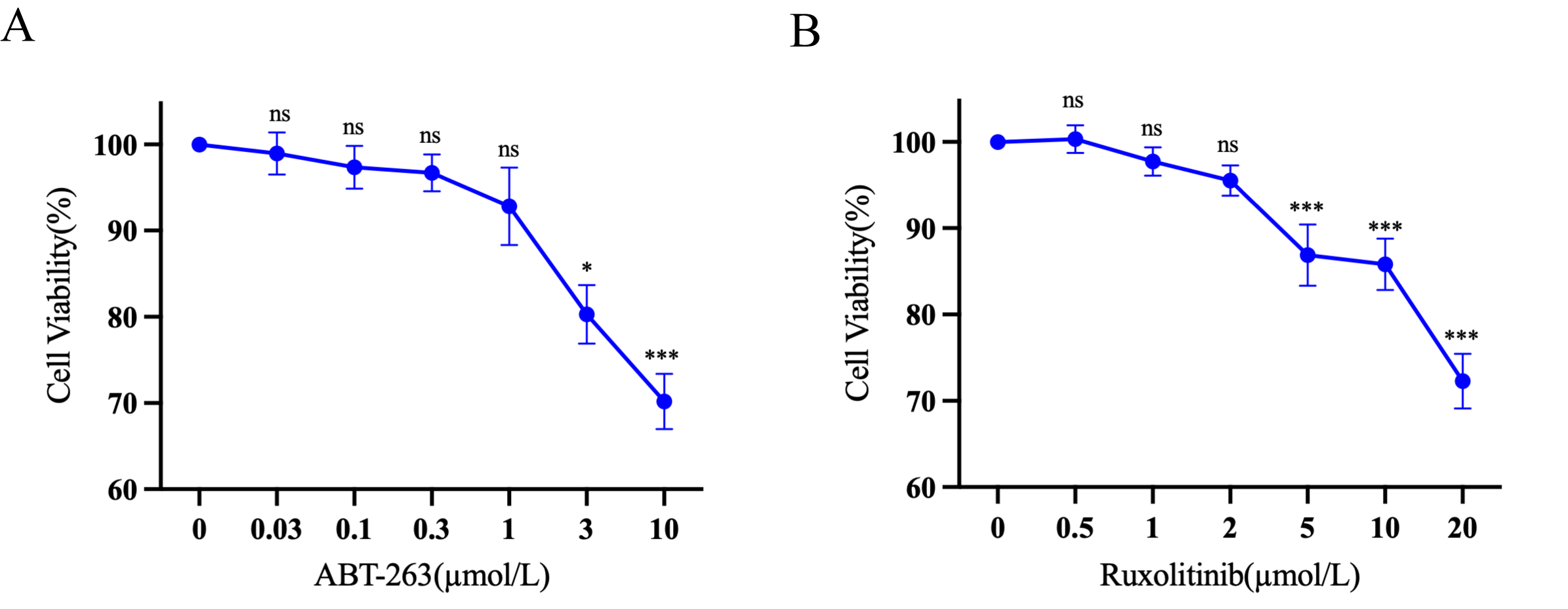


**Supplementary Figure 3**. **Effects of ABT-263 and Ruxolitinib on BM-MSC viability.**

CCK8 assay indicating absorbance at 450 nm after treatment of HD BM-MSCs for 3 days with the indicated concentrations of (A) ABT-263 (0, 0.03, 0.1, 0.3, 1, 3, and 10 μM) and (B) Ruxoltinib (0, 0.5, 1, 2.5, 5, 10, and 20 μM) (n = 3). Data are shown as mean ± SD.


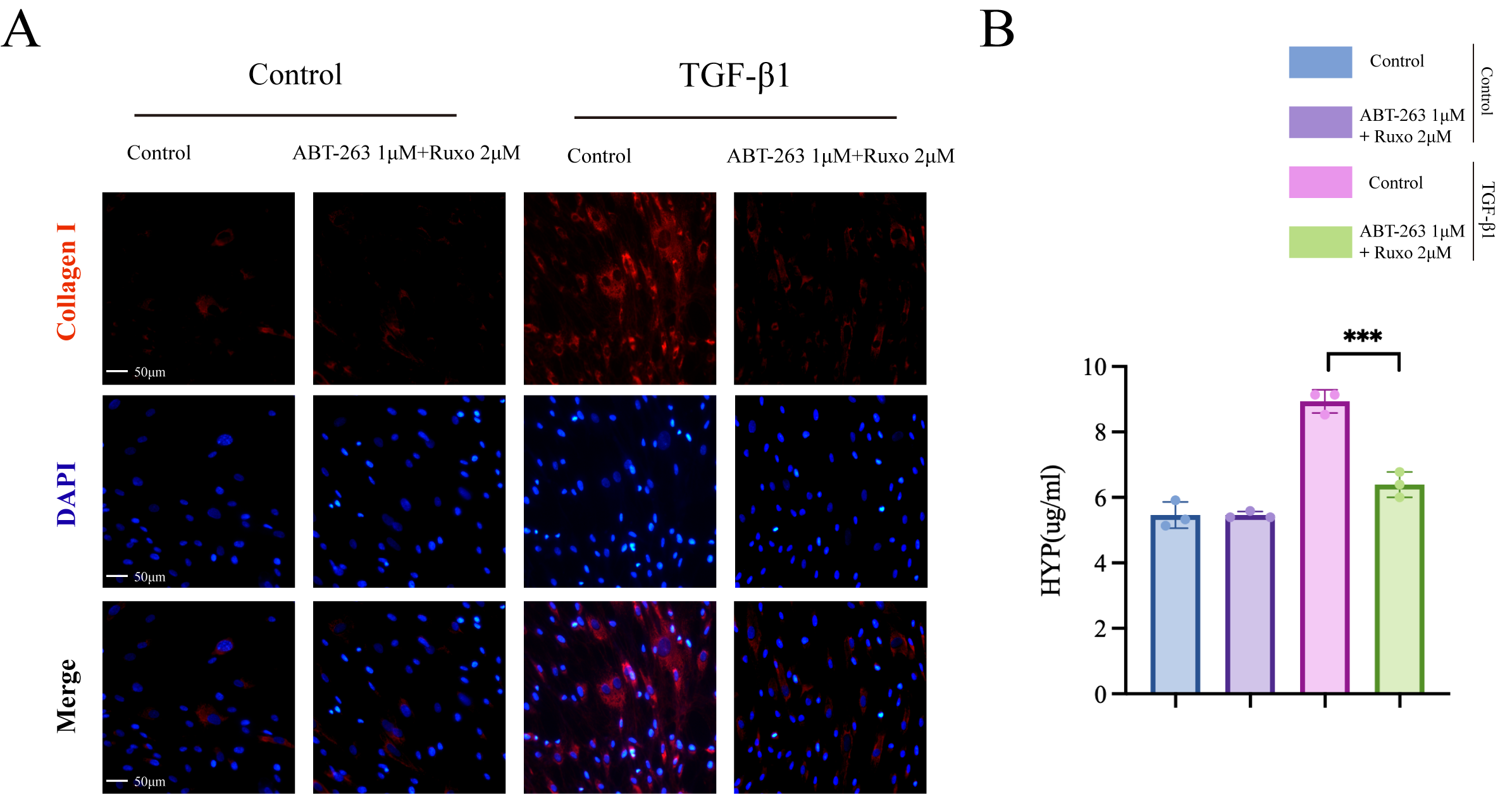


**Supplementary Figure 4**. **Treatment with ABT-263 and ruxolitinib reduces TGFβ-induced fibrosis in BM-MSCs.**

HD BM-MSCs were treated for 3 days with TGF-β1 (5 ng/mL), Ruxolitinib (2 µM), ABT-263 (1 µM), or a combination thereof.

(A) Immunofluorescence staining of Collagen I in BM-MSCs. Collagen I is shown in red, and nuclei are counterstained with DAPI (blue) (n = 3). Scale bar = 25 µm.

(B) Quantification of total collagen content by HYP assay in BM-MSCs. Data are shown as mean ± SD (n = 3). ***p < 0.001.
