# Supplementary material for: BCL-XL drives fibrotic and leukemic progression in myeloproliferative neoplasms
Source: Front Immunol. 2026 Jun 2;17:1818806. doi: 10.3389/fimmu.2026.1818806 (PMC13269428; doi:10.3389/fimmu.2026.1818806)
Supplement: Supplementary file 2 [file DataSheet2.pdf]

Figure1

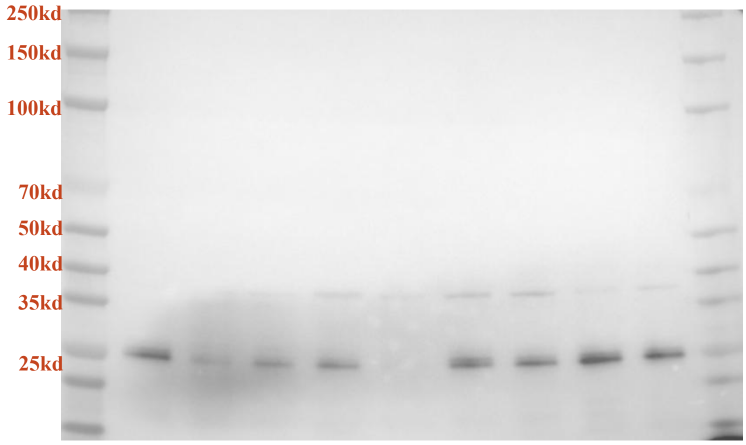

BCL-XL 26KD

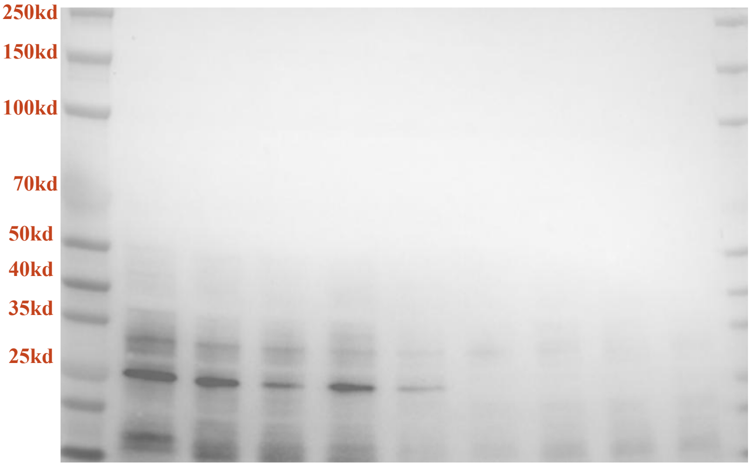

BCL2 26KD

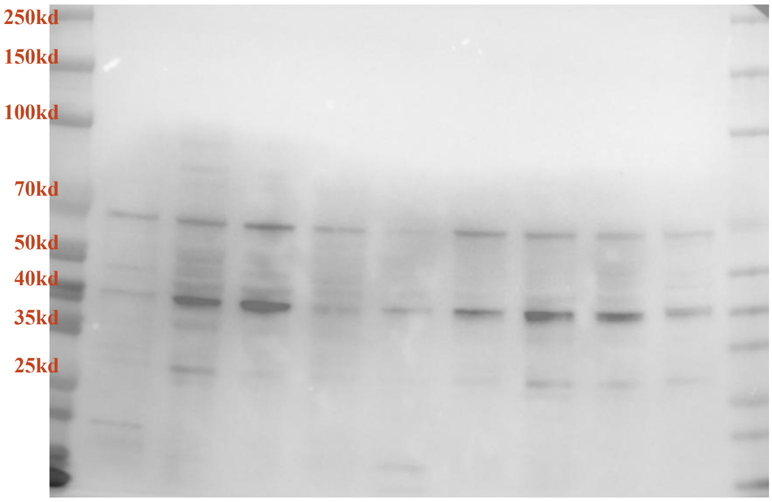

MCL1 40KD

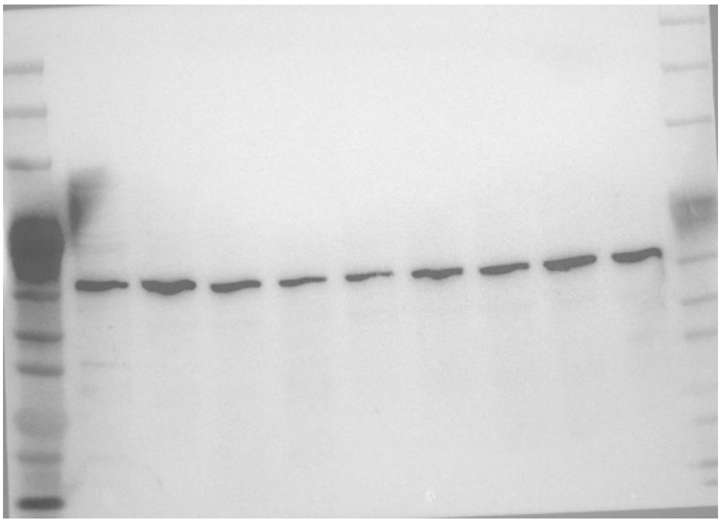

Tubulin 50KD

Figure2

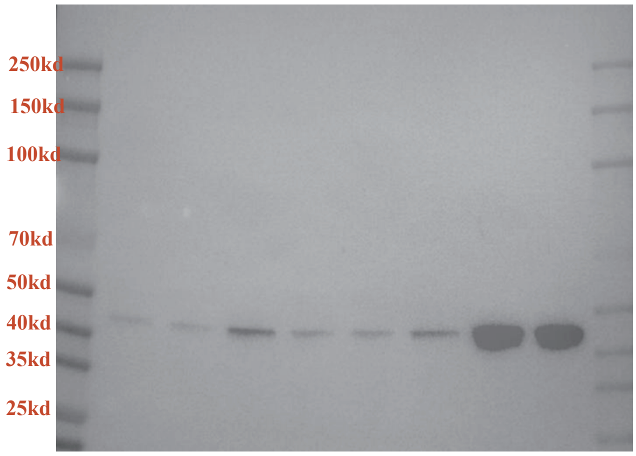

α-SMA 42KD

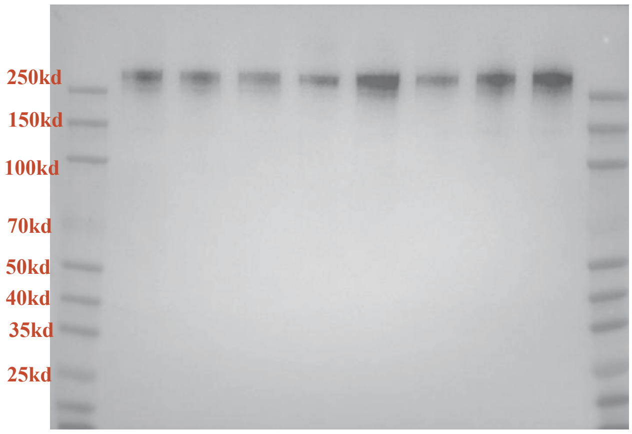

FN 250-275 kDa

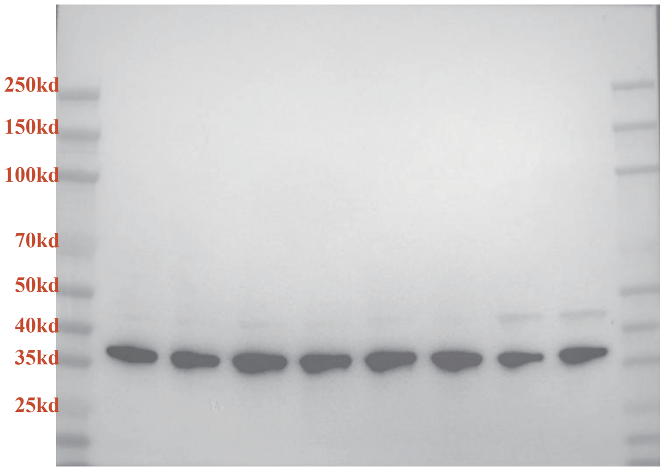

GAPDH 36KD

Figure 3

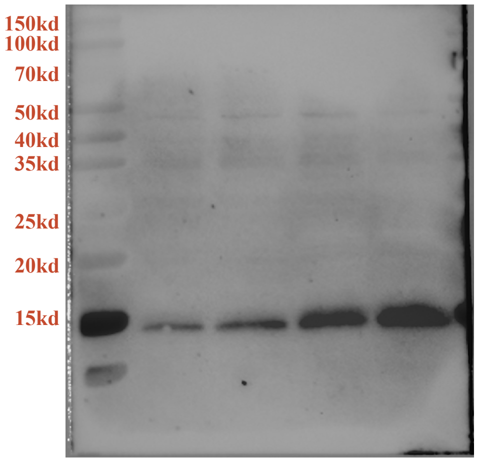

Cytochrome C 12-15KD

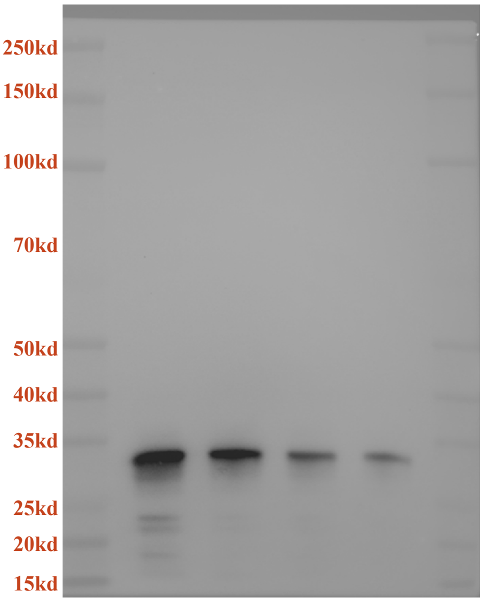

Caspase3 32KD

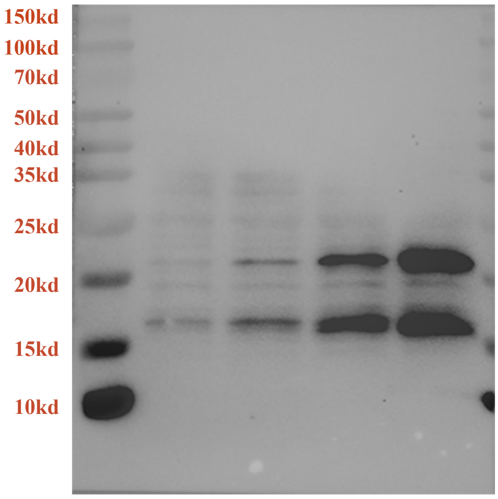

Cleaved-caspase3 17-19KD

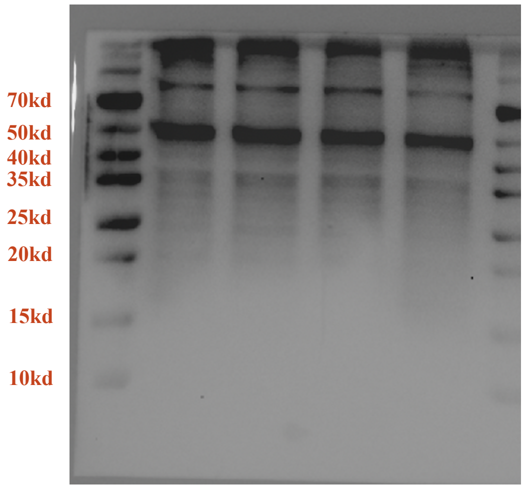

Tubulin 50KD

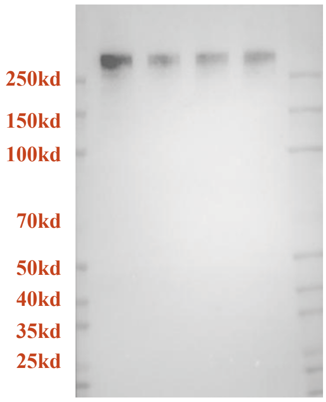

FN 250-275 kDa

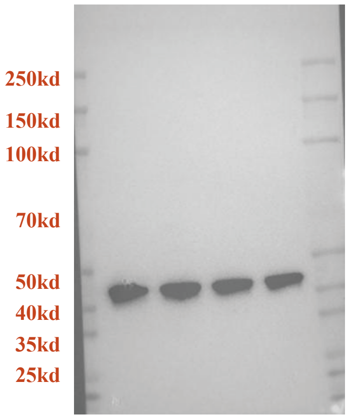

α-SMA 42KD

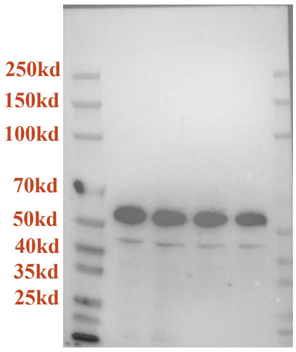

Tubulin 50KD

Figure 4

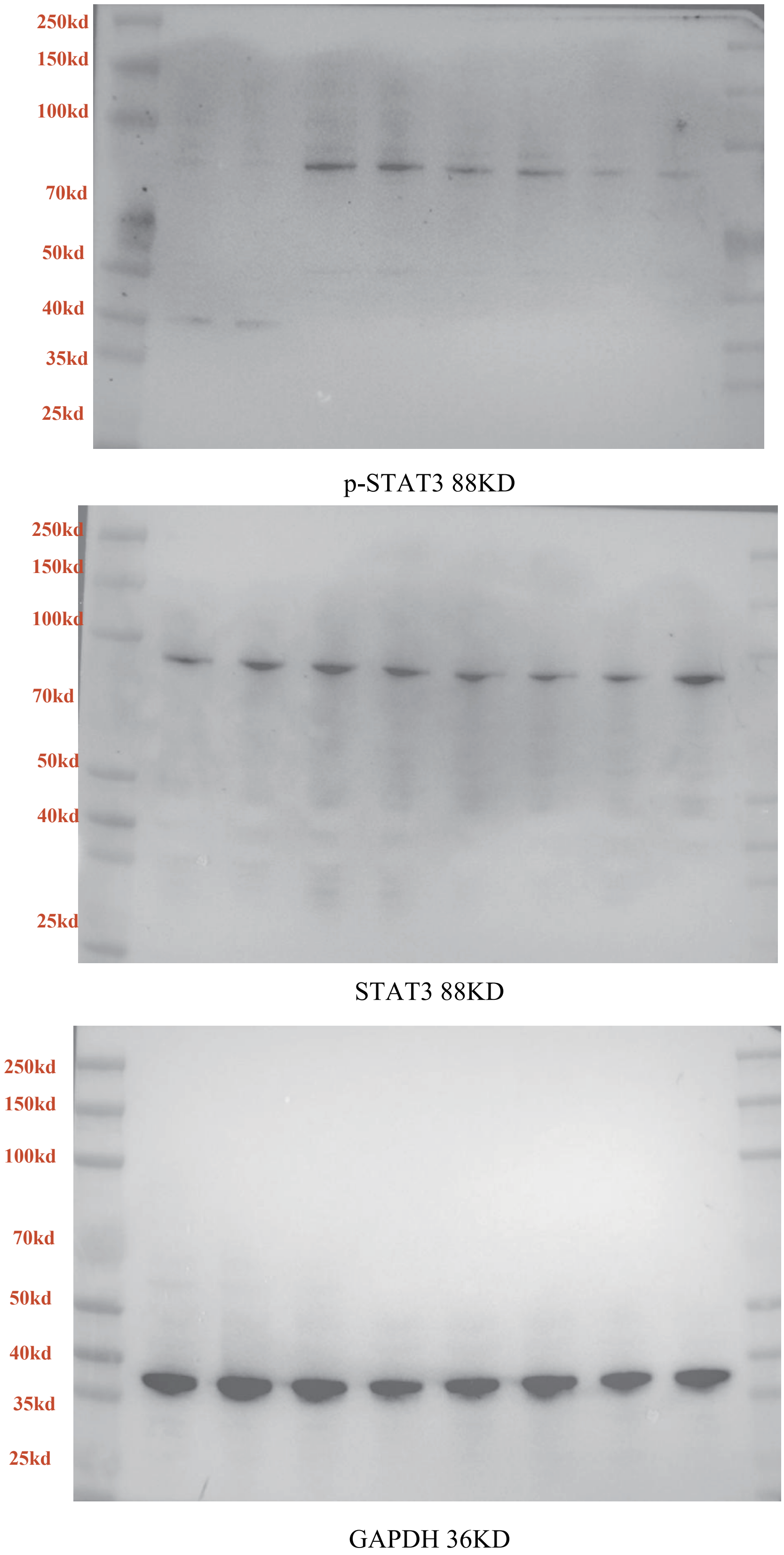
250kd
150kd
100kd
70kd
50kd
40kd
35kd
25kd
p-STAT3 88KD
250kd
150kd
100kd
70kd
50kd
40kd
25kd
STAT3 88KD
250kd
150kd
100kd
70kd
50kd
40kd
35kd
25kd
GAPDH 36KD

Figure 4

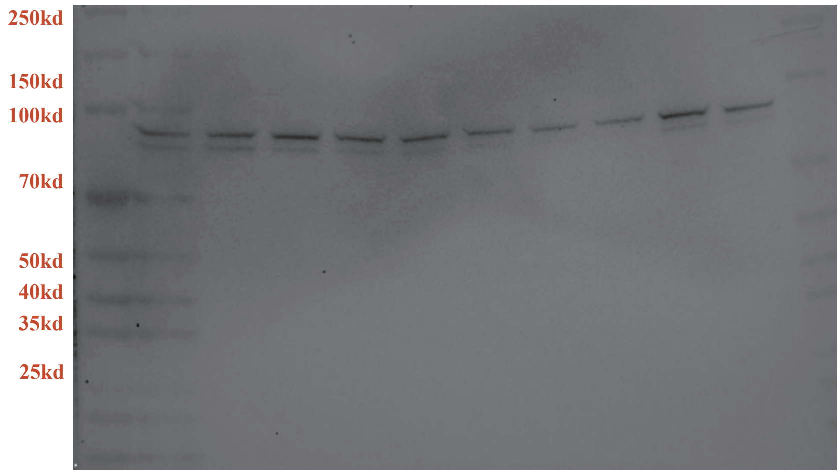

p-STAT3 88KD

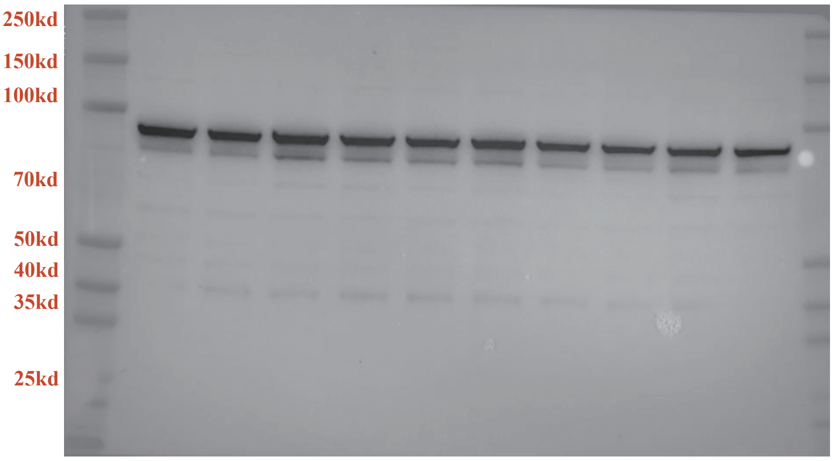

STAT3 88KD

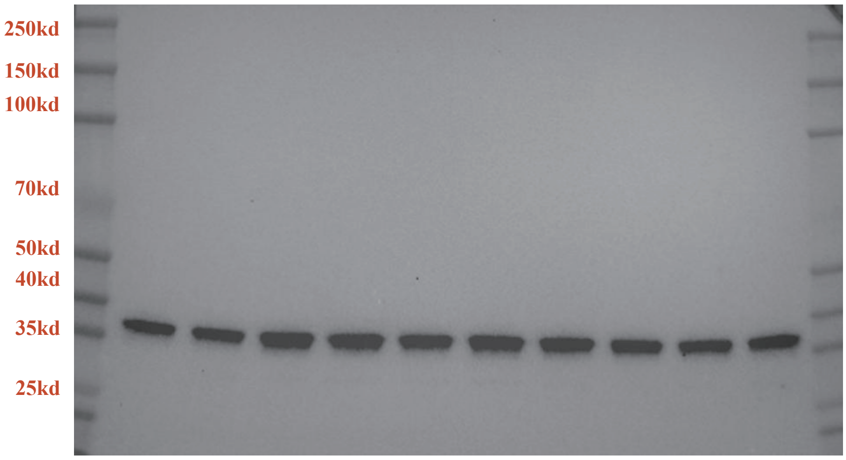

GAPDH 36KD

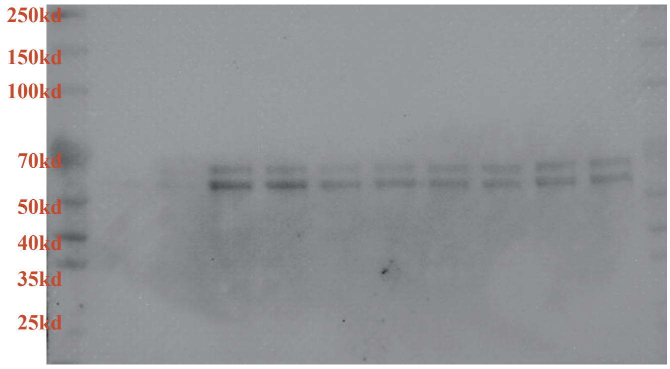

p-SMAD3 46-52KD

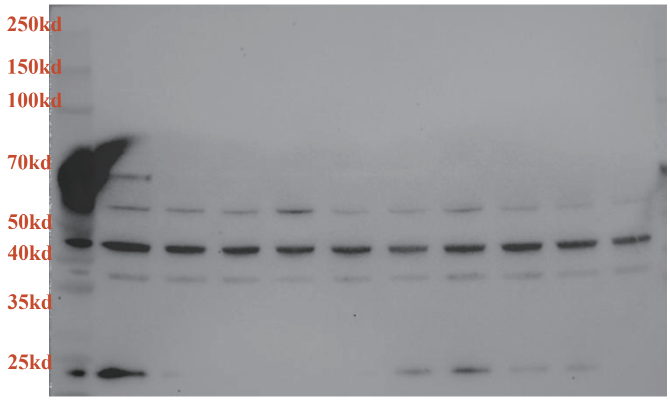

SMAD3 46-52KD

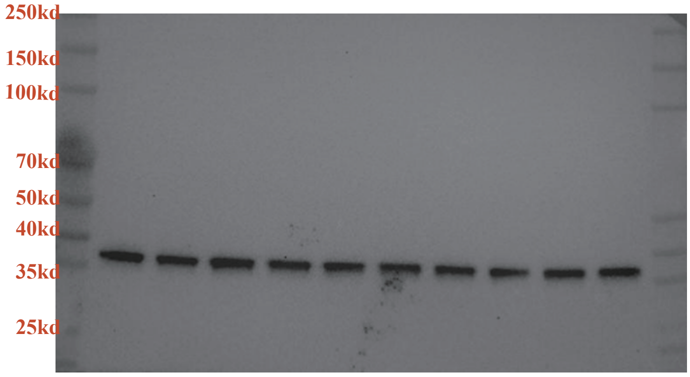

GAPDH 36KD

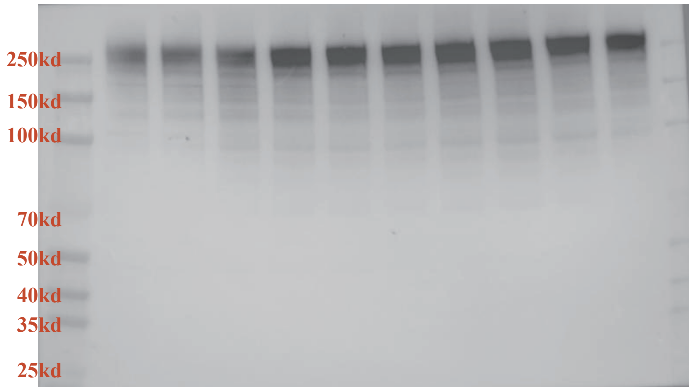

FN 250-275 kDa

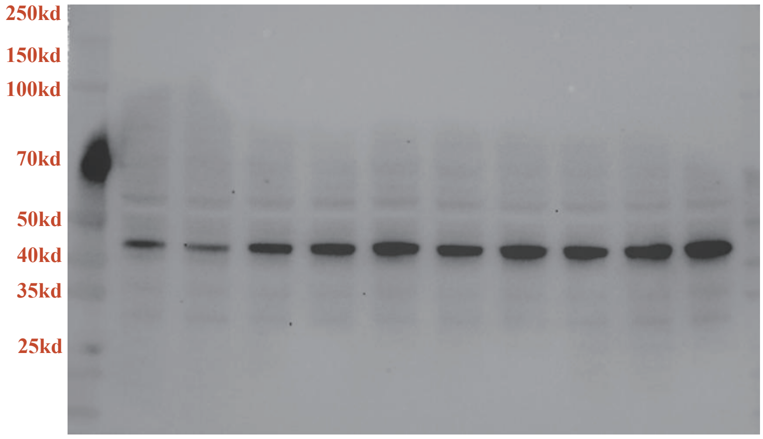

α-SMA 42KD

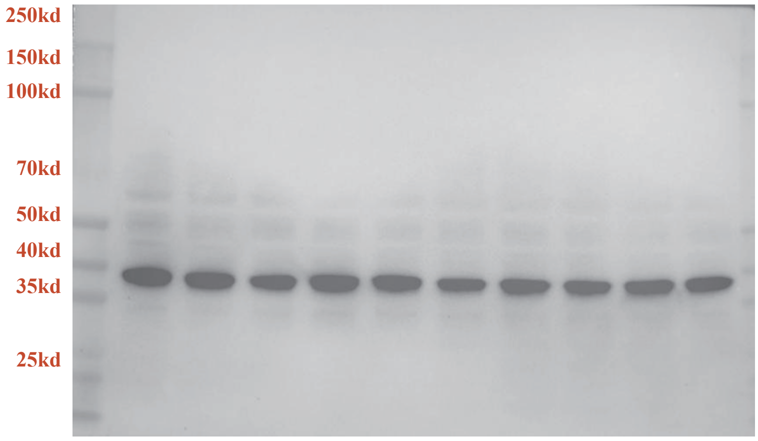

GAPDH 36KD

Figure 5

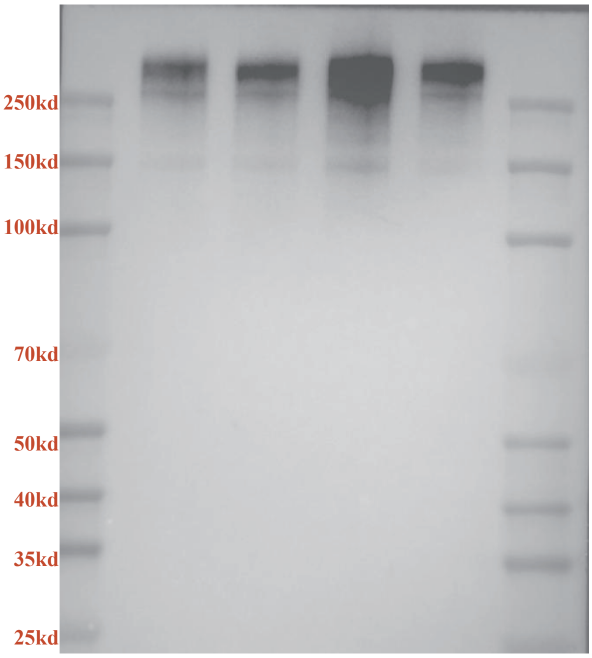

FN 250-275 kDa

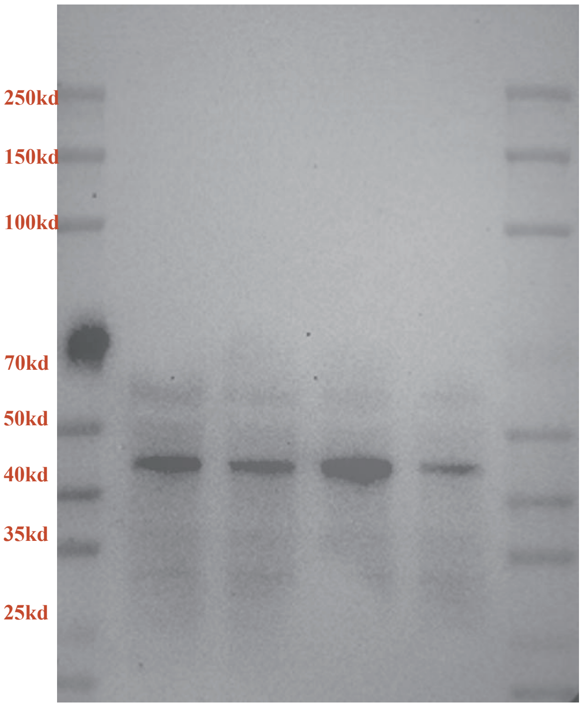

α-SMA 42KD

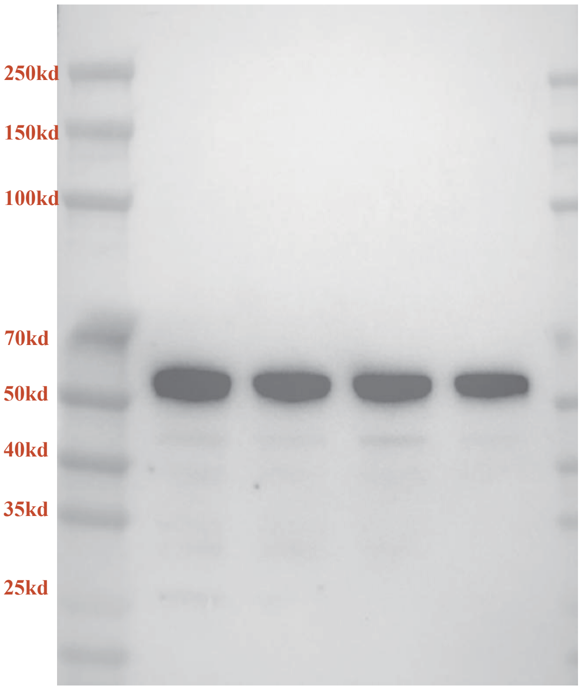

Tubulin 50KD

Figure 6

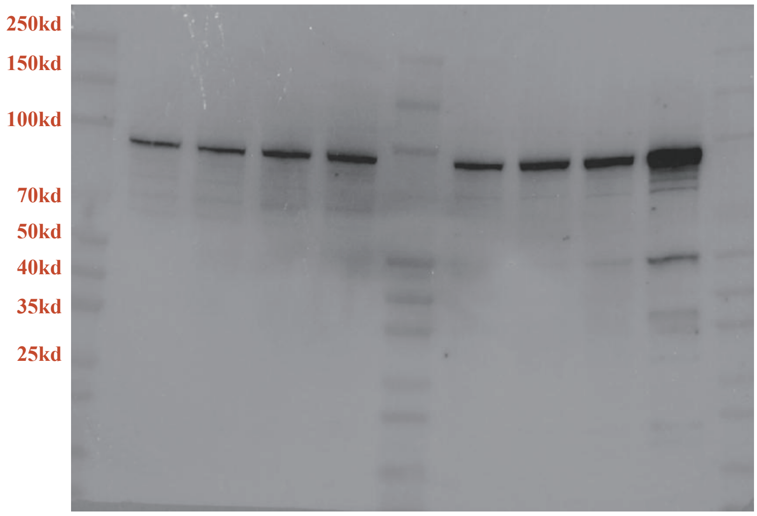

Cleaved-PARP 89KD

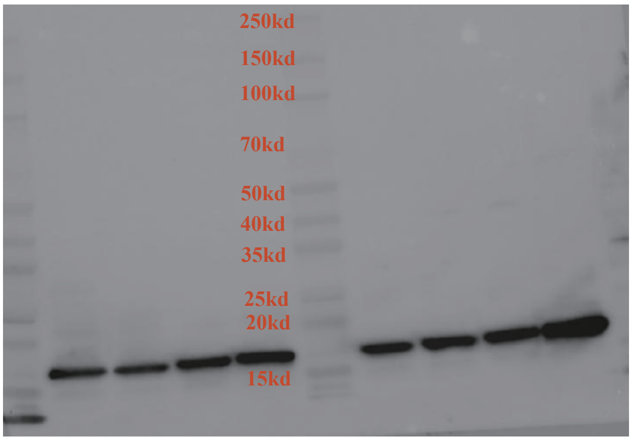

p-H2AX 15KD

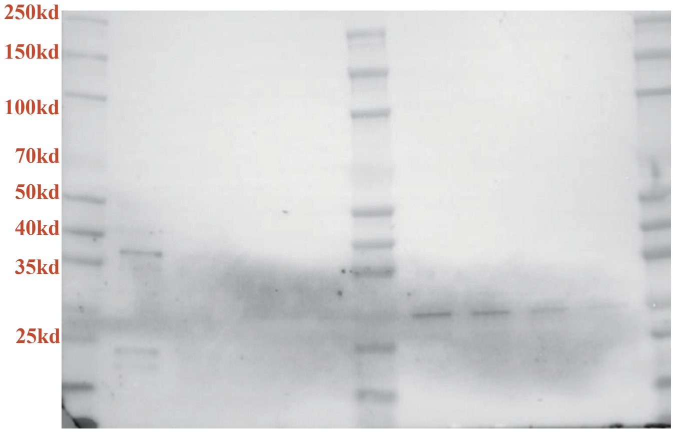

BCL2 26KD

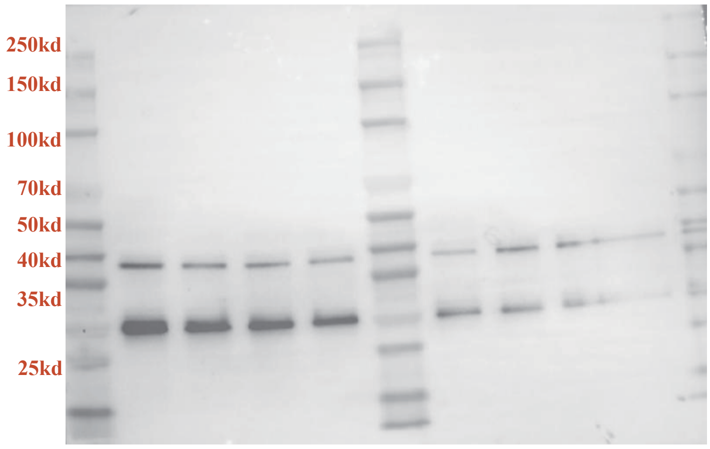

BCLXL 26KD

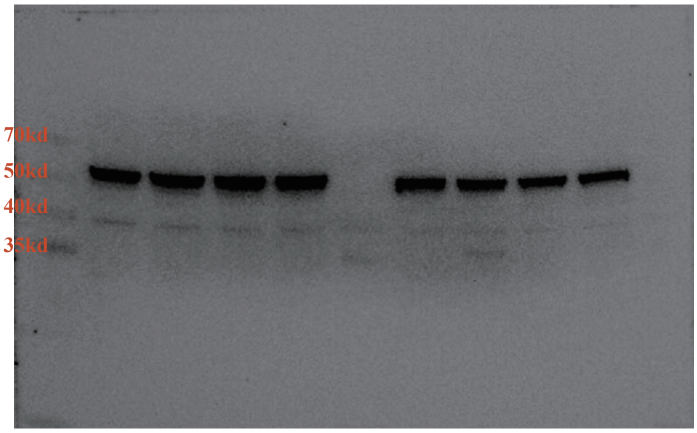

Tubulin 50KD

Figure 7

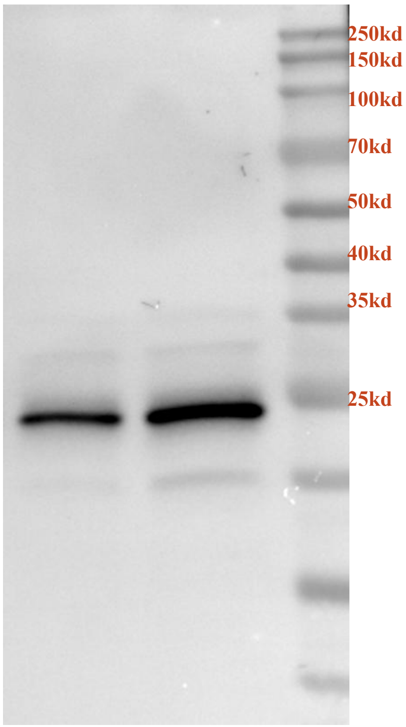

BCL-XL 26KD

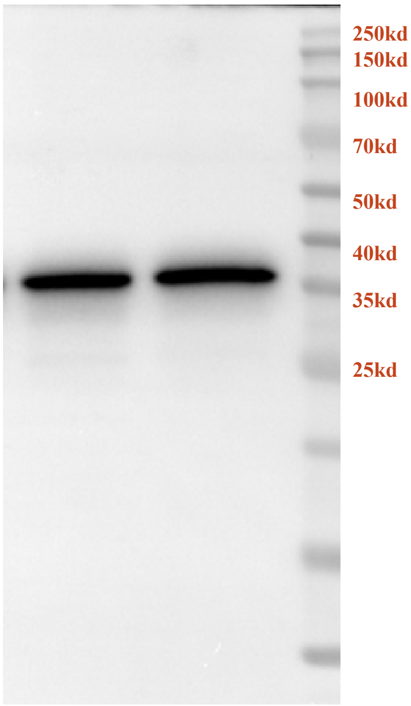

GAPDH 36KD

Supplementary Figure 2

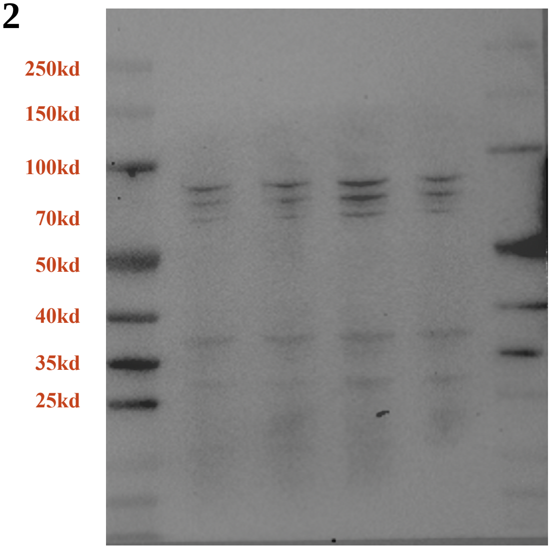

pSTAT3 88KD

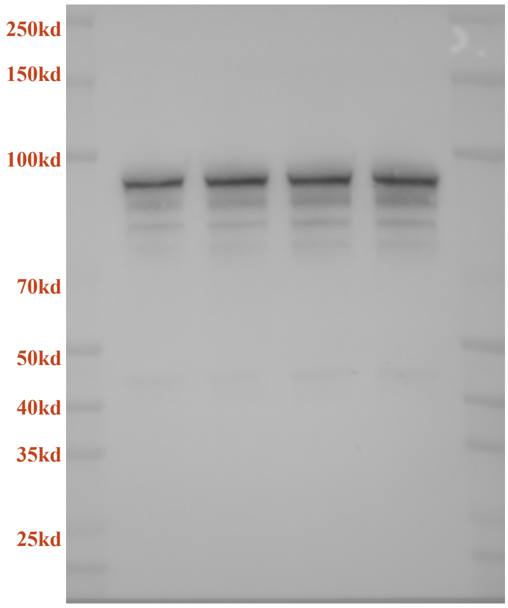

STAT3 88KD

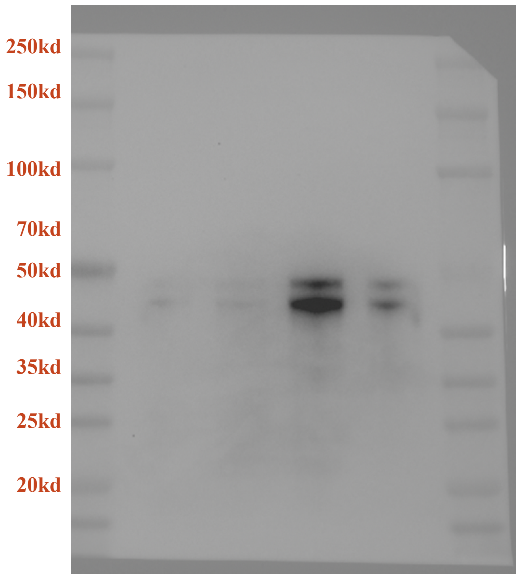

pSMAD3 46-52KD

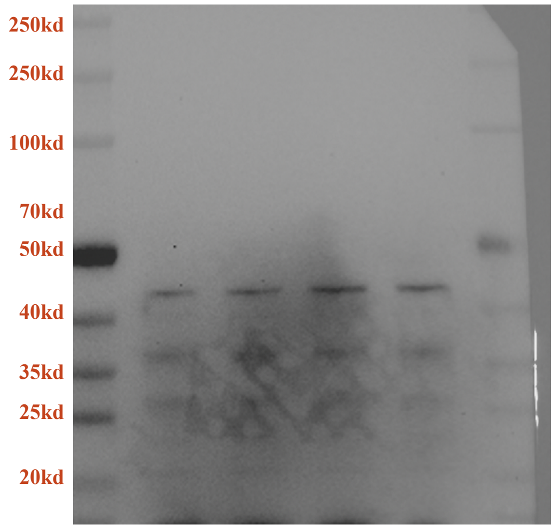

SMAD3 46-52KD

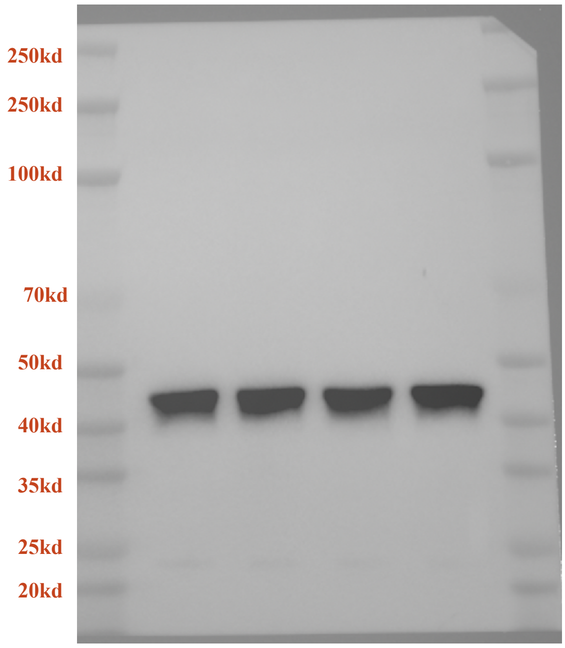

β-actin 40KD

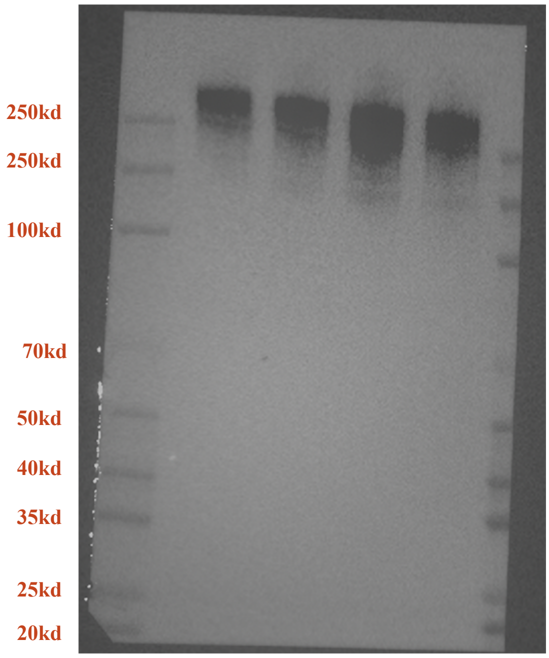

FN 250-275KD

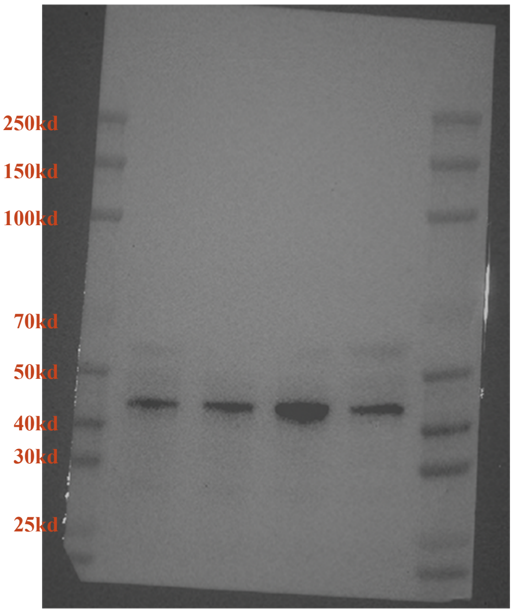

α-SMA 42kd

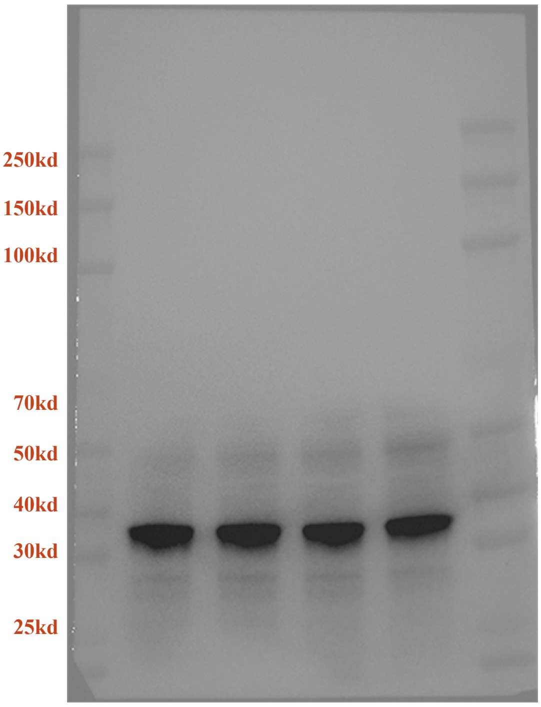

GAPDH 36kd
