# Supplementary material for: BCL-XL drives fibrotic and leukemic progression in myeloproliferative neoplasms
Source: Front Immunol. 2026 Jun 2;17:1818806. doi: 10.3389/fimmu.2026.1818806 (PMC13269428; doi:10.3389/fimmu.2026.1818806)
Supplement: Supplementary file 3 [file Table1.docx]

**Supplementary Table 1**. Characteristics of patients with polycythemia vera (PV), essential thrombocythemia (ET), and primary myelofibrosis (PMF)

| **Patients#** | **Diagnosis** | **Age** | **Gender** | **JAK2 mutation** | **Gradus of fibrosis** |
| --- | --- | --- | --- | --- | --- |
| 1 | ET | 73 | F | + | 0 |
| 2 | ET | 61 | F | + | 0 |
| 3 | ET | 77 | M | + | 0 |
| 4 | ET | 59 | F | - | 0 |
| 5 | ET | 72 | M | + | 1 |
| 6 | ET | 28 | F | - | 0 |
| 7 | ET | 62 | M | + | 0 |
| 8 | ET | 26 | M | + | 0 |
| 9 | ET | 54 | M | + | 1 |
| 10 | ET | 39 | F | - | 0 |
| 11 | PV | 39 | M | + | 0 |
| 12 | PV | 71 | F | + | 0 |
| 13 | PV | 64 | M | + | 0 |
| 14 | PV | 41 | M | + | 1 |
| 15 | PV | 85 | F | + | 1 |
| 16 | PV | 59 | M | + | 0 |
| 17 | PV | 58 | F | + | 0 |
| 18 | PV | 75 | M | + | 0 |
| 19 | PV | 24 | M | + | 0 |
| 20 | PV | 63 | F | + | 1 |
| 21 | PMF | 70 | M | + | 1 |
| 22 | PMF | 70 | M | + | 1 |
| 23 | PMF | 54 | M | + | 1 |
| 24 | PMF | 72 | M | + | 1 |
| 25 | PMF | 75 | M | + | 2 |
| 26 | PMF | 59 | M | - | 1 |
| 27 | PMF | 62 | M | - | 1 |
| 28 | PMF | 72 | F | + | 1 |
| 29 | PMF | 81 | F | + | 3 |
| 30 | PMF | 27 | M | + | 1 |

**Supplementary Table 2:**

| **Name** | **Vendor** | **Cat#** | **Clone** | **Amount (per test)** |
| --- | --- | --- | --- | --- |
| APC anti-human CD90 (Thy1) | BioLegend | #328114 | 5E10 | 5 µL |
| PE anti-CD105 (Endoglin) | BioLegend | #800504 | SN6h | 5 µL |
| FITC anti-human CD73 (Ecto-5'-nucleotidase) | BioLegend | #344016 | AD2 | 5 µL |
| Brilliant Violet 421™ anti-human CD45 | BioLegend | #368522 | 2D1 | 5 µL |
| Zombie Aqua™ Fixable Viability Kit | BioLegend | #423101 | - | 5 µL |

Antibodies and viability dye used in flow cytometry.

**Supplementary Table 3:**

| **Name** | **Vendor** | **Cat#** | **WB** | **IHC** | **ICC/IF** |
| --- | --- | --- | --- | --- | --- |
| FN | Abcam | #ab2413 | 1:1000 | 1:200 | 1:200 |
| α-SMA | Proteintech | #14395-1-AP | 1:1000 | 1:4000 | 1:800 |
| Collagen I | ABclonal | #A24112PM | 1:10000 | - | 1:500 |
| BCL-XL | Proteintech | #10783-1-AP | 1:5000 | 1:250 | - |
| BCL2 | Proteintech | #12789-1-AP | 1:5000 | 1:2000 | - |
| MCL1 | Proteintech | #16225-1-AP | 1:2000 | 1:250 | - |
| Cytochrome C | Proteintech | #10993-1-AP | 1:5000 | - | - |
| Cleaved-caspase3 | Proteintech | #25128-1-AP | 1:1000 | - | - |
| Tubulin | Proteintech | #11224-1-AP | 1:10000 | - | - |
| β-actin | Proteintech | #66009-1-Ig | 1:5000 | - | - |
| GAPDH | Proteintech | #60004-1-Ig | 1:5000 | - | - |
| Caspase3 | Abcam | #ab184787 | 1:2000 | - | - |
| pSTAT3 | Abcam | #ab76315 | 1:2000 | - | - |
| STAT3 | Abcam | #ab68153 | 1:1000 | - | - |
| pSMAD3 | Abcam | #ab52903 | 1:2000 | - | - |
| SMAD3 | Abcam | #ab40854 | 1:5000 | - | - |
| Goat anti-mouse IgG-HRP | [ABKBio](https://abkbio.net/uploadfiles/2025/04/20250423085254309.pdf) | #ABK0020W | 1:10000 | - | - |
| Goat anti-rabbit IgG-HRP | [ABKBio](https://abkbio.net/uploadfiles/2025/04/20250423085254309.pdf) | #ABK0021W | 1:10000 | - | - |
| ABflo®555-anti-rabbit IgG (H+L) | ABclonal | #AS058 | - | - | 1:200 |
| ABflo® 488-anti-rabbit IgG (H+L) | ABclonal | #AS053 | - | - | 1:200 |

Detailed information on antibody suppliers, catalog numbers, and applications.
